# Immuno-Oncology: Emerging Targets and Combination Therapies

**DOI:** 10.3389/fonc.2018.00315

**Published:** 2018-08-23

**Authors:** Henry T. Marshall, Mustafa B. A. Djamgoz

**Affiliations:** Neuroscience Solutions to Cancer Research Group, Department of Life Sciences, Imperial College London, London, United Kingdom

**Keywords:** combination immunotherapy, tumor microenvironment, checkpoint blockade, personalized therapy, biomarkers

## Abstract

Host immunity recognizes and eliminates most early tumor cells, yet immunological checkpoints, exemplified by CTLA-4, PD-1, and PD-L1, pose a significant obstacle to effective antitumor immune responses. T-lymphocyte co-inhibitory pathways influence intensity, inflammation and duration of antitumor immunity. However, tumors and their immunosuppressive microenvironments exploit them to evade immune destruction. Recent PD-1 checkpoint inhibitors yielded unprecedented efficacies and durable responses across advanced-stage melanoma, showcasing potential to replace conventional radiotherapy regimens. Neverthless, many clinical problems remain in terms of efficacy, patient-to-patient variability, and undesirable outcomes and side effects. In this review, we evaluate recent advances in the immuno-oncology field and discuss ways forward. First, we give an overview of current immunotherapy modalities, involving mainy single agents, including inhibitor monoclonal antibodies (mAbs) targeting T-cell checkpoints of PD-1 and CTLA-4. However, neoantigen recognition alone cannot eliminate tumors effectively *in vivo* given their inherent complex micro-environment, heterogeneous nature and stemness. Then, based mainly upon CTLA-4 and PD-1 checkpoint inhibitors as a “backbone,” we cover a range of emerging (“second-generation”) therapies incorporating other immunotherapies or non-immune based strategies in synergistic combination. These include targeted therapies such as tyrosine kinase inhibitors, co-stimulatory mAbs, bifunctional agents, epigenetic modulators (such as inhibitors of histone deacetylases or DNA methyltransferase), vaccines, adoptive-T-cell therapy, nanoparticles, oncolytic viruses, and even synthetic “gene circuits.” A number of novel immunotherapy co-targets in pre-clinical development are also introduced. The latter include metabolic components, exosomes and ion channels. We discuss in some detail of the personalization of immunotherapy essential for ultimate maximization of clinical outcomes. Finally, we outline possible future technical and conceptual developments including realistic *in vitro* and *in vivo* models and inputs from physics, engineering, and artificial intelligence. We conclude that the breadth and quality of immunotherapeutic approaches and the types of cancers that can be treated will increase significantly in the foreseeable future.

## Introduction

**Figure 1:**
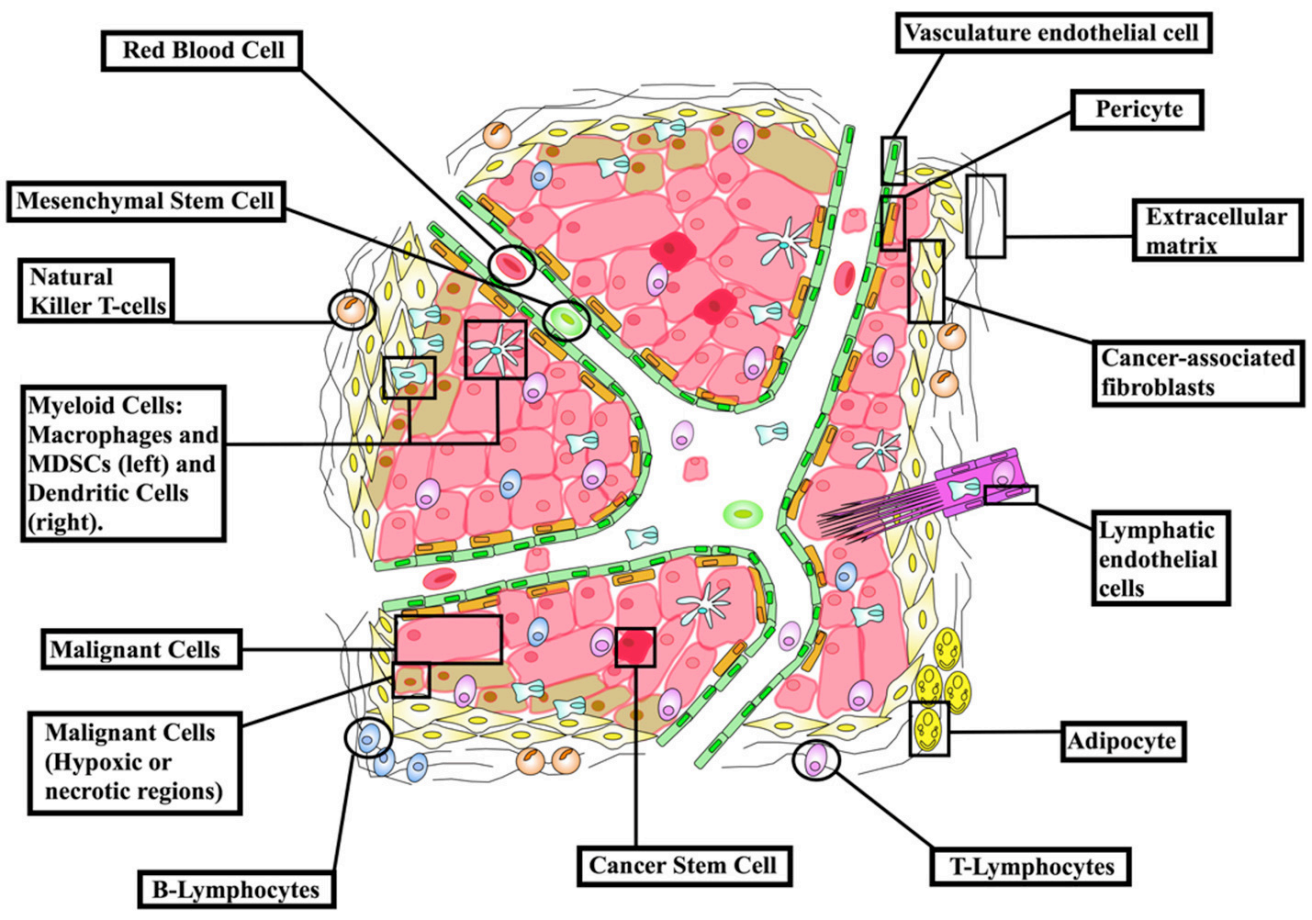
The cellular make-up of the tumor microenvironment (TME). The tumor niche possesses a dynamic structural topography with significant spatial variability in vascular supply, growth factor and cytokine accessibility, ECM-derived structural support and interactions with immune cells. TME hence contributes to tumor heterogeneity as a “rogue organ,” formed by normal-malignant cell associations. Created using information from Balkwill et al. (2) and Tang et al. (3).

Non-surgical treatments of cancer (mainly conventional chemotherapy, targeted biological therapies, and radiotherapy) have not generated completely satisfactory results to date. The ongoing problems include low target selectivity, drug resistance, inability to effectively address metastatic disease and severe side effects. In contrast, immunotherapies that overall provoke host immunity to induce a systemic response against tumors currently offer much clinical promise. Although most malignant tumors can be recognized by the host immune-surveillance defensive system, namely natural killer (NK) and T-cells, cancer cells evolve to acquire genetic instabilities and other associated “hallmarks” that can enable immune evasion and persistent growth (1). Host immunity has been shown to detect tumor cell “neoantigens” *in vitro*. However, neoantigen recognition alone cannot eliminate tumors *in vivo* given the inherent complex micro-environment, heterogeneous nature and stemness of tumors (Figure 1) (2, 3). Indeed, neoantigens are seldom recognized and spontaneously elicit T-cell antitumor responses (4).

**Figure 2:**
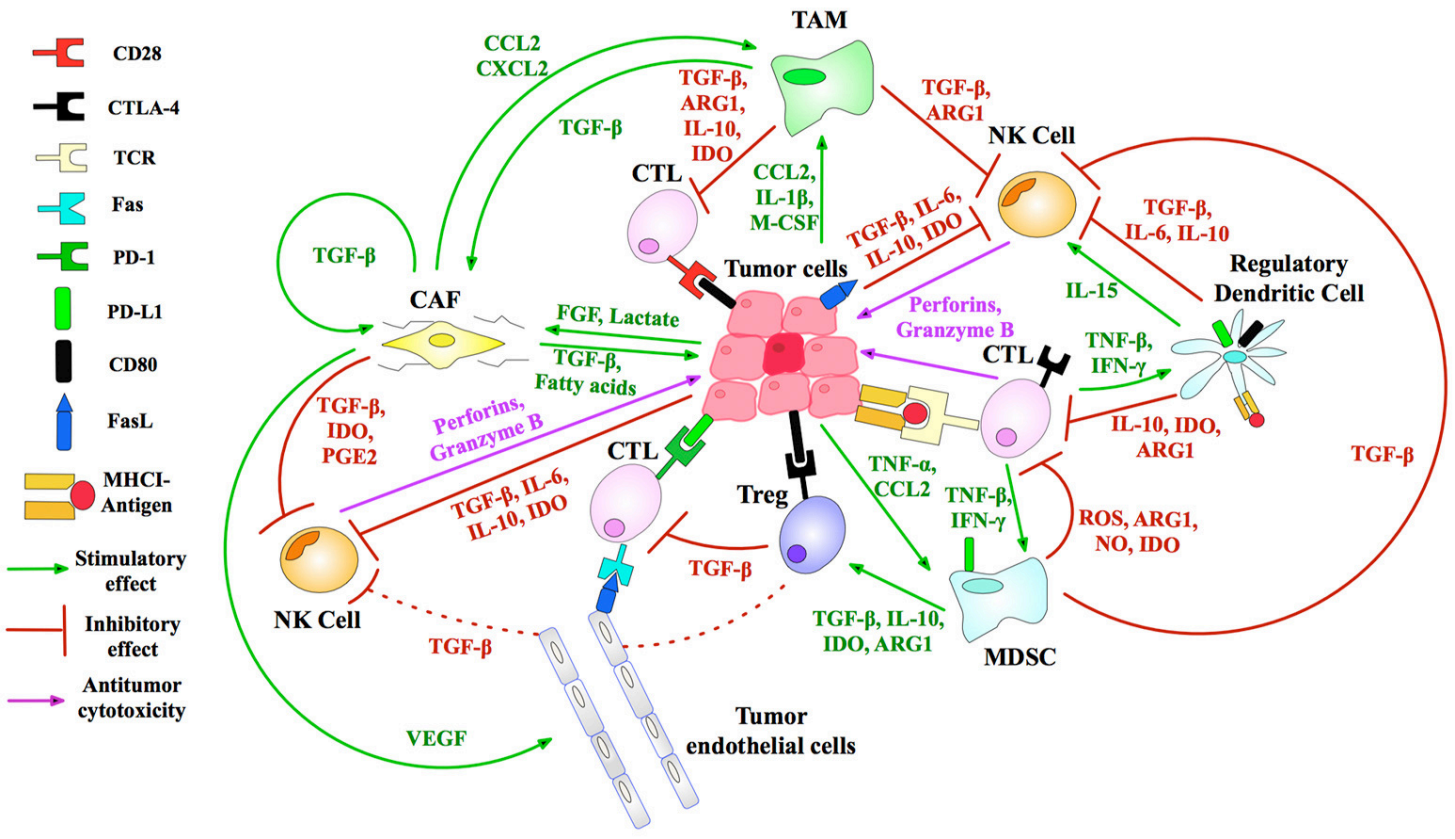
Immunosuppressive mechanisms of the TME. Treg (regulatory T-) cells generate IL-10 and TGF-β angiogenic cytokines to suppress CTL (cytotoxic T-lymphocyte) activity. Myeloid-derived suppressor cells (MDSCs) produce reactive oxygen species (ROS), arginase (ARG) and nitric oxide (NO) that inhibit T-cell activation. Tumor-associated macrophages (TAMs) similarly block CTL and natural killer (NK) T-cells, immature dendritic cells cause T-cell anergy via IDO enzyme secretion, while cancer-associated fibroblasts (CAFs) and endothelial cells (tumor, lymphatic, and vascular) produce TGF-β and stimulate T-cell apoptosis by FasL-Fas binding (5, 6). MHC I is downregulated in tumor cells to inhibit T-cell recognition. FasL is expressed by tumors, killing T-cells (7). Tumors secrete VEGF to sustain tumor endothelial cells, and lactate and FGF to promote CAF development (8). Immunosuppressive TAMs are maintained by a suite of tumor secretions: CCL2, CXCL12, and IL-1β (8). NK cell inhibition by tumors is accomplished by release of IL6/10, IDO, and TGF-β. CAFs suppress NK cells via cytokines and growth factors including PGE2, TGF-β, and IDO (6). Tumors recruit immunosuppressive to the TME via TNF-α and CCL2 (9). IDO, indoleamine 2,3-dioxygenase; CD80, cluster of differentiation 80; M-CSF, macrophage colony-stimulating factor; CCL2, chemokine ligand 2; PGE2, prostaglandin E2; CXCL2, chemokine (C-X-C motif) ligand 2; TGF, transforming growth factor; IL, interleukin. Figure created by combining information from Jeanbart and Swartz (5), Hargadon et al. (10), Derbal et al. (8), Hasmim et al. (6), and Baginska et al. (9). See Abbreviations list for further definitions.

An array of normal immune cells, including T-cells, B-cells, and NK cells, together with endothelia, associate with cancer cells and extracellular matrix to form the tumor micro-environment (TME) (Figure 2). This is a dynamic immunosuppressive network and a major obstacle to immunotherapeutic intervention (3). Within TME, adipocytes, regulatory T (Treg) cells, and fibroblasts, along with a network of cytokines and growth factors, promote cellular proliferation across all stages of tumorigenesis. Thus, both malignant and non-malignant components of tumors, as well as the mediators of their intercellular communication, are potential targets for immunotherapy (2).

**Figure 3:**
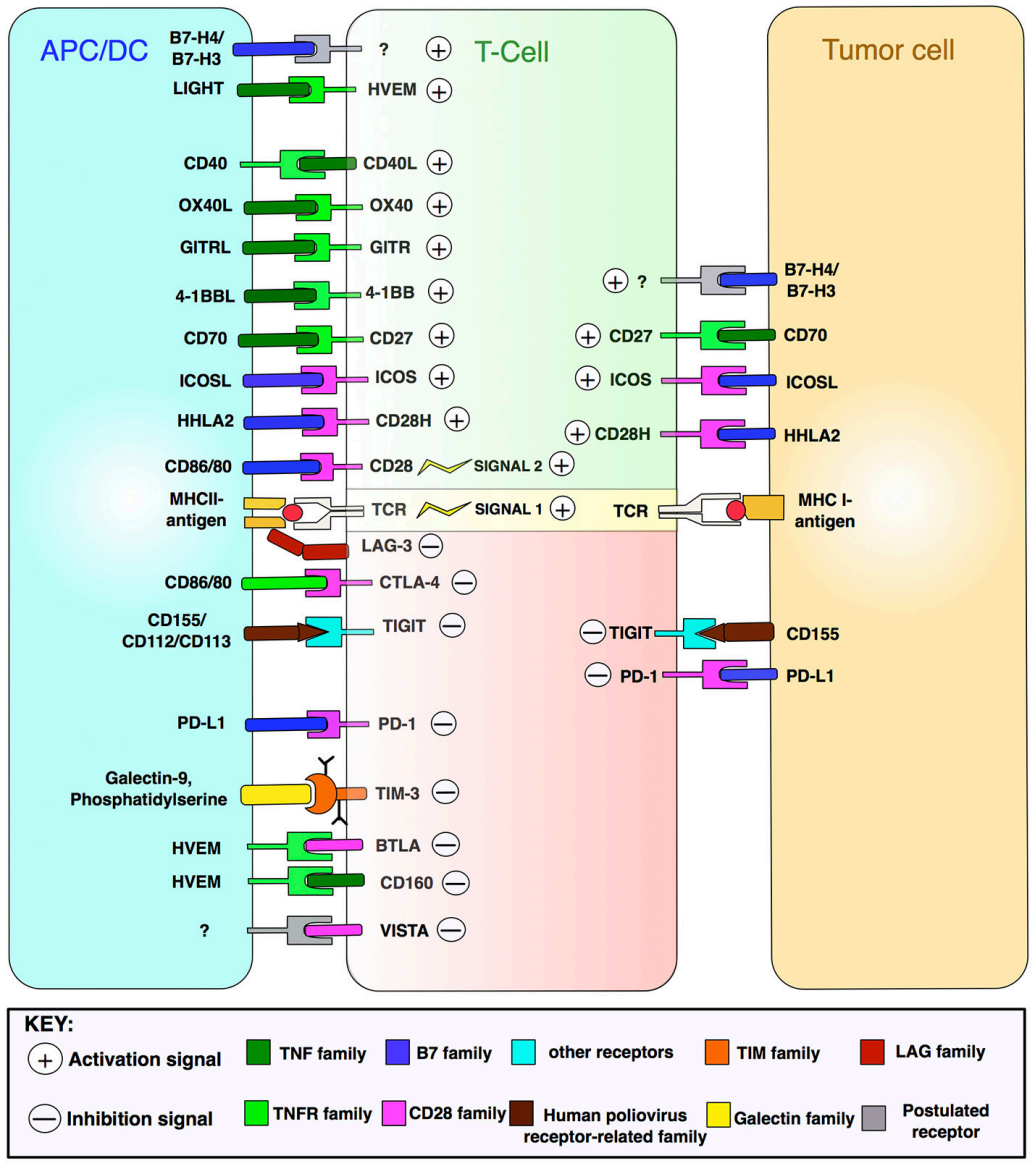
T-cell activation and cell-surface therapeutic targets. T-cell activation by APC/DCs and impact upon the tumor cell is driven by many integrated signals. Depicted are immune receptor-ligand pairings amenable to pharmacological manipulation by immunomodulatory mAbs. HVEM, herpes virus-entry mediator; LIGHT, lymphocyte activation gene 3 protein; GITR, glucocorticoid-induced TNFR family-related protein; ICOS, inducible T-cell costimulatory; LAG-3, lymphocyte activation gene 3 protein; TIGIT, T-cell immunoreceptor with Ig and ITIM domains; TIM-3, T-cell Ig mucin domain-containing 3; BTLA, B-lymphocyte and T-lymphocyte attenuator; VISTA, V-domain Ig suppressor of T-cell activation; TNF, tumor necrosis factor. Figure created by combining information from Mahoney et al. (13), Melero et al. (14), and Khalil et al. (15). See Abbreviations list for further definitions.

**Figure 4:**
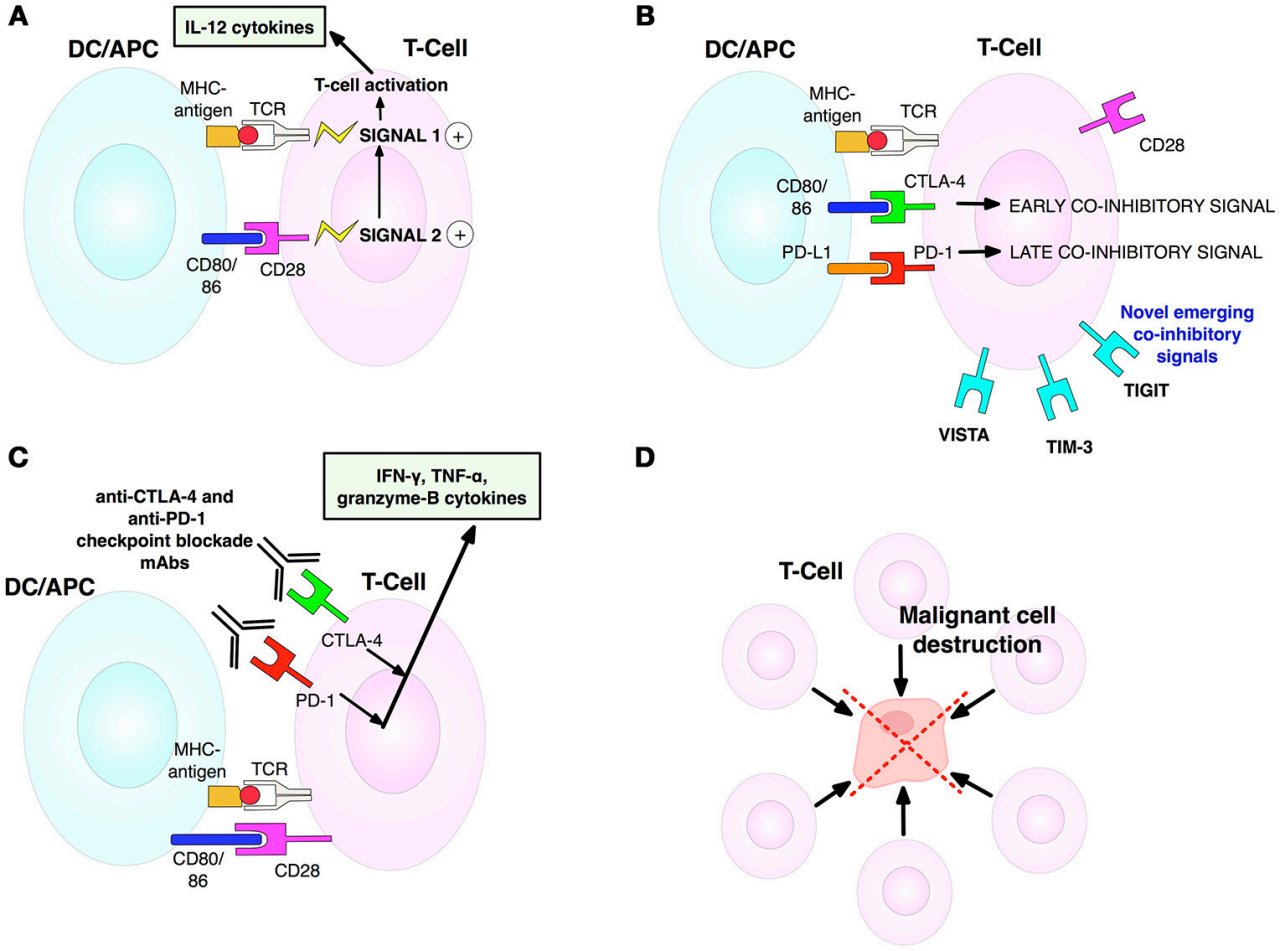
T-cell activation, inhibition and anti-CTLA-4/anti-PD-1 blockade mechanisms. **(A)** T-cell activation is initiated by TCR-MHCI-antigen interaction (signal 1). Full activation and effector activity demand additional CD28-CD80/86 binding (signal 2). Both signals cause T-cells to secrete IL-2 that drives T-cell proliferation and differentiation. **(B)** T-cell activation is limited by CTLA-4, upregulated on activated T-cells. CTLA-4 outcompetes CD28 for CD80/86 ligands, thus stopping signal 2 needed for T-cell activation. Contrarily, later coinhibitory PD-1 checkpoint interacts with its ligand to diminish T-cell cytotoxic activity in tumors expressing PD-L1. **(C)** Dual checkpoint anti-CTLA-4/PD-1 blockade mAbs block inhibitory CTLA-4 and PD-1 checkpoints, enabling release of cytokines involved in sustaining activated T-cells. CD28 can now bind its ligand to enable signal 2. **(D)** Activated T-cells can now join the antitumor T-cell effector response to destroy tumor cells. Adapted from Mellman et al. (17). See Abbreviations list for further definitions.

**Figure 5:**
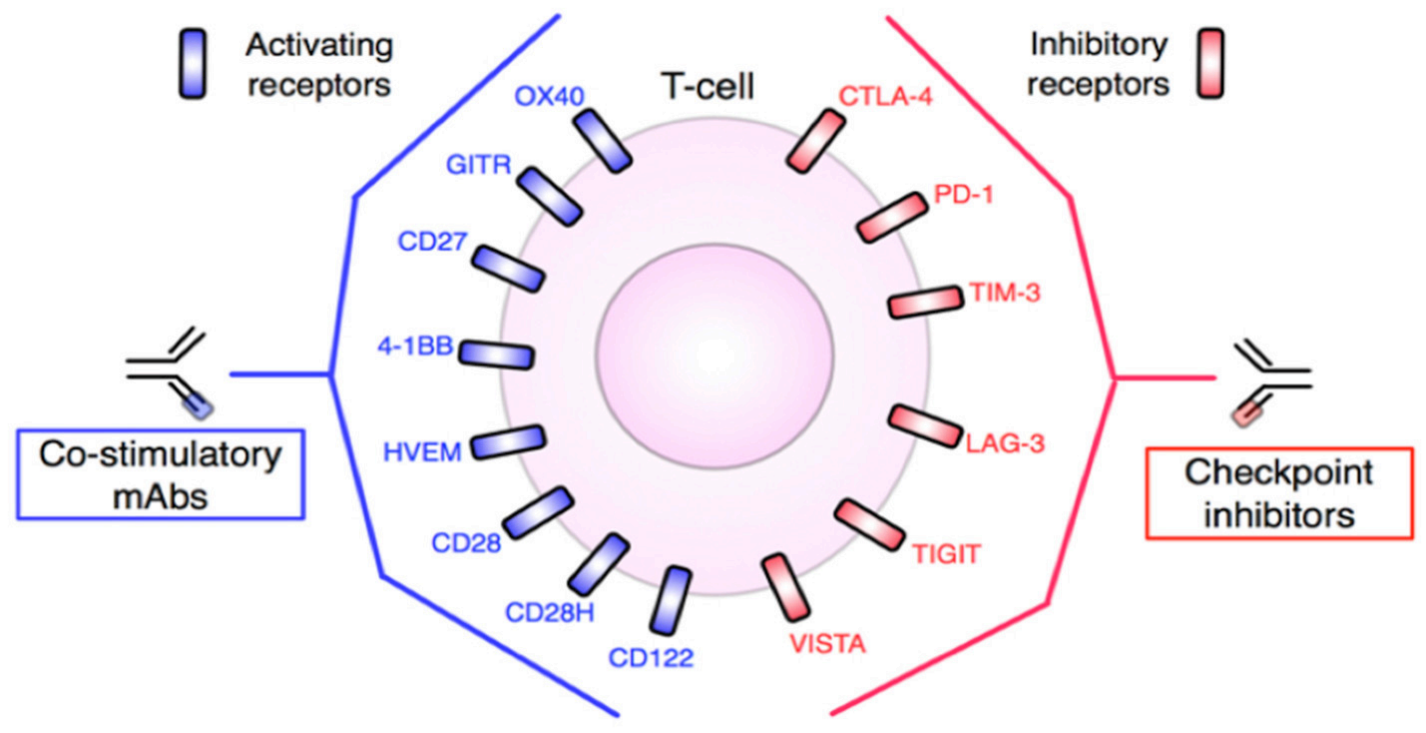
T-cell targets for mAb-based immunotherapy. Inhibitory and stimulatory receptors expressed in the TME may be targeted for therapeutic intervention. Agonistic antibodies, such as anti-OX40 or anti-CD28, target and activate co-stimulatory molecules, while blocking or antagonist antibodies, including anti-PD-1 or anti-CTLA-4, block T-cell inhibitory molecules. In either case, T-cells are stimulated and tumor destruction promoted. Adapted from Mellman et al. (17) and Vasaturo et al. (18). See Abbreviations list for further definitions.

Immune checkpoint receptor pathways represent a major class of “immune synapse,” a cell-cell contact that suppresses T-lymphocyte effector functioning (11). This is likely to be an evolutionary countermeasure against autoimmunity, aiming to minimize damage to uninfected cells in virus-infected tissues and to limit systemic inflammation (12). However, tumors can exploit these mechanisms to evade immune detection (Figure 3) (12, 16). Hence, such mechanisms provide opportunities for immunotherapy intervention (Figures 4, 5) (19). A plethora of such therapies are currently in preclinical development and clinical application. These include T-cell immune receptor modulating monoclonal antibodies (mAb's), vaccines, adoptive cellular therapy (ACT), engineered oncolytic viruses (OVs), small-molecule targeting drugs, and cytokine-based adjuvant therapies (Table 1). Checkpoint inhibitors, both as monotherapies and in combination, have generated some of the most significant therapeutic efficacies at least in subpopulations of cancer patients (15, 13, 22). Notably, proof-of-principle has been provided for checkpoint inhibitor mAb's, e.g., anti-CTLA-4 (ipilimumab/Yervoy) and anti-PD-1 (nivolumab/Opdivo and pembrolizumab/Keytruda) (13, 22). Compared with conventional therapies, these drugs demonstrated significantly higher efficacy and durability as well as reduced toxicity. Importantly, also, a broad spectrum of malignancies could be targeted (19, 22, 23).

**Table 1:** Selected single-agent cancer immunotherapies (15, 20, 21).

**Therapeutic modality**	**General usage**	**Current limitations**	**Example**	**Development status**
mAbs	Very selective agonism (costimulatory mAbs) or antagonism/blockade (checkpoint inhibitors) of immune receptor-ligand pairings.	Very expensive—over $120,000 per monotherapy. Time demanding manufacture/development. Challenges in determining ideal treatment timing and duration. Need to minimize “on-target, off-tumor” effects. Often cancer-specific. Need to identify more optimal combinations.	Immune checkpoint blockades/inhibitors: anti-CTLA-4, anti-PD-1, anti-PD-L1, anti-LAG3. Costimulatory mAbs: anti-GITR, anti-OX40, anti-CD40.	Anti-PD-1 (nivolumab and pembrolizumab) and anti-CTLA-4 (ipilimumab) mAbs are FDA approved for melanoma. Many others entering clinical trials for NSCLC, RCC and kidney cancers.
Vaccines	Cancer vaccines introduce tumor-specific antigens to be taken up by dendritic or antigen presenting cells that in turn prime and boost the T-cell antitumor immune response.	Many are poorly immunogenic on their own and require adjuvants to generate effective immune responses, but these adjuvants often increase toxicity. Low response rates of ~20–30%, so need to identify patient subgroups with a specific cancer and develop personalized therapies.	Vaccines targeting gp100, MUC1, MAGE-A3. Cvac, BiovaxID, hepcortespenlisimut-L, and Neuvenge are currently in development.	FDA-approved vaccines include: Oncophage for kidney cancer, and sipuleucel-T for metastatic prostate cancer. Many are entering clinical development, and several e.g., BiovaxID are in phase III trials.
Small molecules	Uniquely specialized for specific intracellular targets, but also suitable for extracellular or cell surface targets.	Off-target activity/lack of specificity, dose-limiting toxicity, not effective at blocking immune protein-protein interactions. Often demands daily administration.	Inhibitors of VEGF (bevacizumab), HDAC (entinostat), DNMT (5-azacytidine). TKIs (imatinib), BRAF mutant inhibitor (vemurafenib). IDO1 and COX2 inhibitors.	IDO, HDAC, DNMT, VEGF, and TK inhibitors are in clinical trials. Several efficacy trial results are awaited.
Adoptive T-cell therapy	Tumor-targeted cytotoxicity against both intracellular and extracellular tumor-specific antigens.	Tumor heterogeneity - mutated antigen expression and composition impacts response rates. “On-target, off-tumor” toxicity issue. Ultimately, need to translate deep remissions into cures by fine-tuning regimens and targeting many antigens simultaneously. Commercial mass production difficult.	Tumor-infiltrating lymphocytes (against mutated EBR2) for bile duct cancer, genetically engineered CARs (against CD19) for lymphoma and genetically engineered T-cells with sTCRs (against MART-1) for melanoma.	Clinical trials have been accelerating since 2010, with over 20 clinical trials in progress against melanoma, lymphoma and leukemias.
Cytokines	Agonism or antagonism of immune protein-protein pathways.	Generally high toxicities. Poor pharmacokinetics.	GM-CSF, IL-12, IL-15, IL-21, IFN-γ, and TNF-α.	IL-2 is now approved for metastatic melanoma and RCC. IFN-alpha approved for stage III melanoma.
Oncolytic viruses	Exploits viral ability to replicate and kill tumor cells while simultaneously stimulating patient-specific antitumor immune responses.	Need to better characterize mechanisms of action, develop combinatorial immunotherapies, and scale-up for mass commercialization.	Talimogene laherparepvec or T-VEC used for melanoma.	T-VEC became FDA-approved in 2015 as Imlygic for patients with inoperable cancers. Other phase I trials are ongoing for pancreatic, breast and colorectal cancers.

In this review, we explore emerging trends in immunotherapy that are at various stages of development. First, we give an overview of current immunotherapy modalities. Then, we give an account of emerging “next-generation” immune checkpoints and combination immunotherapies. In particular, the latter has surged in popularity since the reports of significantly enhanced treatment efficacy obtained using dual checkpoint blockade with ipilimumab + nivolumab, compared to either drug alone. We also consider the importance of predictive and prognostic biomarkers, including PD-L1, to stratify tumors, boost clinical trial efficacy and increase patient response. Finally, we highlight several categories of promising novel targets that could further enhance the effectiveness of immunotherapy.

## Current immunotherapy modalities: an overview

Immune checkpoint inhibitors currently represent the most promising cancer therapeutics where even monotherapies can produce durable responses in 40-50% of patients, persisting long after treatment has ceased (Table 1) (24, 25). The main strategies are those stimulating effector mechanisms and those neutralizing immunosuppressive mechanisms (16). Vaccine-based oncotherapy using tumor antigen infusion enhances the innate anti-tumor ability of a patient's immune system (26). Additional stimulatory approaches administer genetically engineered OVs to initiate systemic immune responses, use ACT to directly deliver immune cells into patients, or apply co-stimulatory mAb's specific to members of the tumor necrosis factor receptor (TNFR) superfamily to bolster T-cell function. Immunosuppressive tumor mechanisms include checkpoint inhibitor mAb's targeting inhibitory T-cell checkpoints of PD-1 and CTLA-4, and other targeted antibodies (e.g., against CD25) that deplete inhibitory regulatory Treg cells (16).

**Figure 6:**
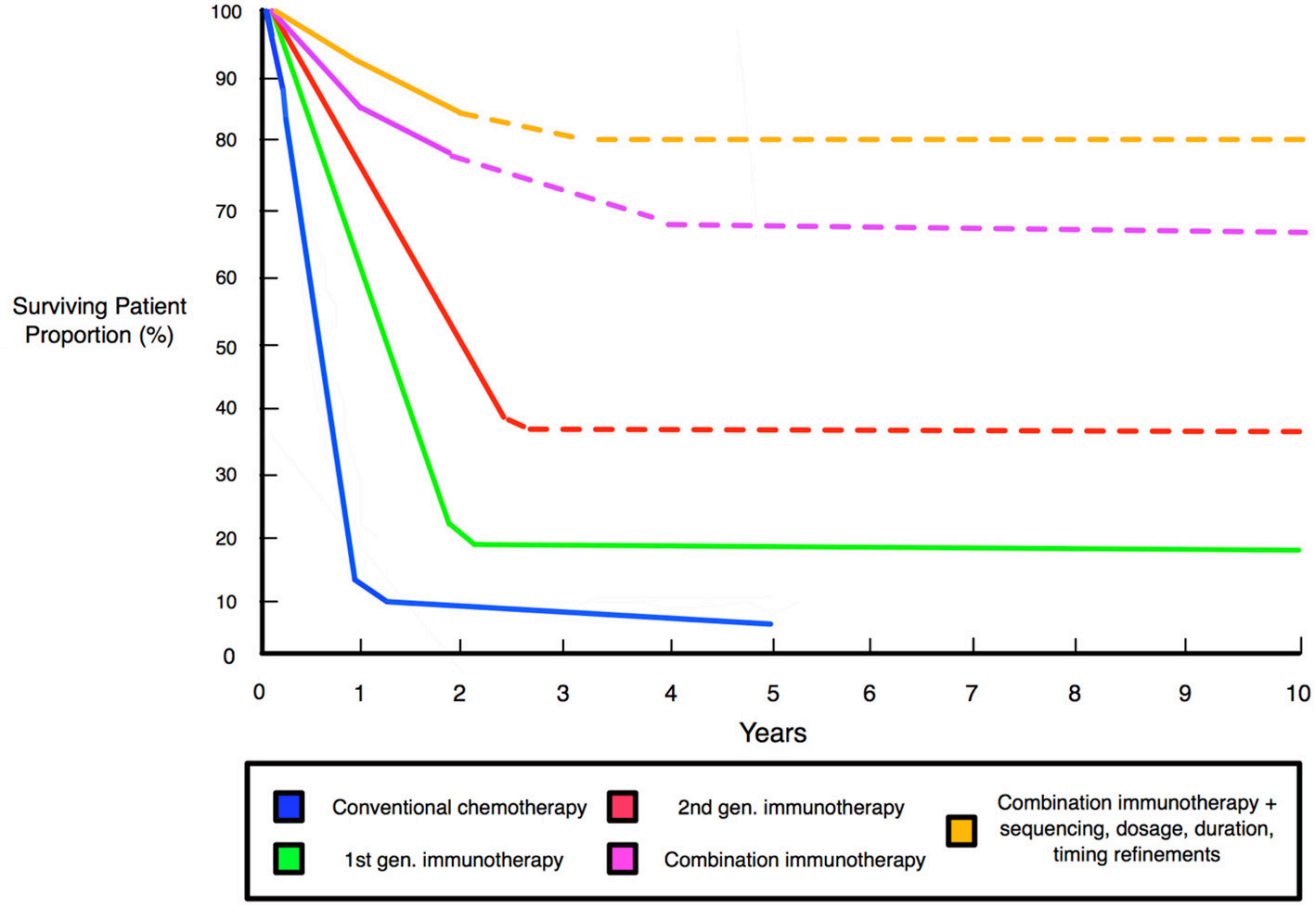
Schematic comparison of patient survival associated with different therapies and improved survival with combination immunotherapy. Graph shows significantly improved survival for immunotherapies relative to conventional chemotherapy. First generation immunotherapies entail anti-CTLA-4 ipilimumab and the therapeutic vaccine Sipuleucel-T that defined the initiating wave of modern immunotherapies. Second generation immunotherapies are exemplified by anti-PD-1 nivolumab and pembrolizumab, and anti-PD-L1 agents of durvalumab and atezolizumab, that deliver effective responses in 40% of patients across many clinical trials (42). Combinations, such as dual-checkpoint CTLA-4/PD-1 blockade, produce strong effects in 60–70% of patients and alongside multifunctional single-agent modalities, represent the “third generation” of immunotherapies (40, 41). Dashed lines indicate projected survival rates based upon preclinical and clinical trials.

Although single-agent immunotherapies, especially checkpoint inhibitors, have demonstrated promising efficacies in some patients with late-stage cancers, however, benefit in most cases was limited (13). In addition, even effective treatments suffered from significant toxicity (3, 25). Checkpoint inhibitors can induce pressing “immune-related adverse effects” (irAEs) due to supra-stimulation of immunity. This could impact upon normal adaptability of vital organs such as liver, heart, kidneys, and pancreas and give rise to type 1 diabetes, pancreatitis, arthritis, and lymphocytic myocarditis (27). Also, autoimmune diseases such as hypophisitis, autoimmune hepatitis, pneumonitis, and inflammatory colitis have been reported frequently with use of nivolumab and ipilimumab (27–32). Thus, risk of immune reactions of healthy organs to checkpoint inhibitors remains an understudied area, and immuno-oncologists must tread a “very fine line” between maximizing anti-tumor efficacy and triggering autoimmunity (27, 33). More seriously, in a study on a mixed cohort of cancer patients, CTLA-4 or PD-1 blockade was found to induce a 2-fold *increase* in tumor development and 50% increase in tumor burden (34). Patients with rare, extra copies of MDM2/4 (“murine double minute 2 homolog”) proto-oncogenes had the greatest risk of such “hyper-progression” (35). In another recent study on a murine model of non-Hodgkin's lymphoma, PD-1 signaling prevented cancerous T-cell proliferation, i.e., PD-1 blockade would actually reactivate cancerous T-cells to promote their replication and hence accelerate malignant growth (36). All these highlight the need for profiling individual cancers and patient genomes for best treatment outcome (34–36). Overall, therefore, there are significant limitations in immunomonotherapies given also the intricate heterogeneity and stemness of human tumors (16). Although corticosteroids and supplementary immunosuppressive therapy can help alleviate undesirable side effects, it is synergistic “combination immunotherapy” that holds the greatest promise (15, 37–39). Combinations simultaneously targeting different components of tumor development/progression can significantly enhance efficacy, response rates, and durability relative to single-agent first- and second-generation immunotherapies (40, 41) (Figure 6). These “third-generation” novel combinations are increasingly based upon the PD-1/PD-L1 blockade “backbone,” given its relatively favorable safety profile and efficacy compared to other checkpoint inhibitors (Table 2) (12, 14, 25, 40, 41). Improved immune targeting and combination therapies owe their enhanced efficacy over monotherapies to the strengthening of multiple components of T-cell anti-tumor responses. This improvement results from (i) functioning of effector T-cells inside TME, including the capacity to evade immunosuppressive checkpoints and soluble factors; (ii) effective extravasation of T-lymphocytes from lymphoid organs into TME; and (iii) production of adequate quantities of effector T-cells inside lymphoid organs (22).

**Table 2:** Selected key combinations of immunotherapies in preclinical and clinical development with checkpoint blockade as “backbone.”

**Combination approach**	**Mechanisms of action and corresponding drug/agent**	**Developmen*t* Stage**	**Tumor type**	**References**
Dual checkpoint blockade	Anti-PD1 (nivolumab) *plus* anti-CTLA-4 (ipilimumab)	I, II, III	Solid tumors	(22, 43, 44)
	Anti-PDL1 (durvalumab) *plus* anti-CTLA-4 (tremelimumab)	Ib, II	Breast, NSCLC	(45, 46)
	Anti-PD1 (pembrolizumab) *plus* anti-CTLA-4 (ipilimumab)	I, Ib	Melanoma, RCC, Lung	(47–49)
	Anti-PD1 (nivolumab) *plus* anti-LAG-3 (BMS-986016)	I/IIa	Solid tumors	(50)
Checkpoint blockade and co-stimulatory receptor agonists	Anti-CD40 (CP-870,863) *plus* anti-CTLA-4 (tremelimumab)	I	Melanoma	(51)
	Anti-CD27 (CDX-1127) *plus* anti-PD1 (nivolumab)	II	ALL	(52)
	Anti-OX40 (MEDI6383) *plus* anti-PDL1 (durvalumab)	I	Solid tumors	(53)
	Anti-GITR (MK-4166) *plus* anti-PD1 (pembrolizumab)	I	Solid tumors	(54)
	Anti-4-1BB (PF-05082566) *plus* anti-PD1 (pembrolizumab)	Ib	Solid tumors	(55)
Bispecific antibodies (BiTEs)	Anti-CD19 (Blinatumomab/MT-103) *plus* dasatinib chemotherapy	II	ALL	(56)
Checkpoint blockade and small molecule inhibitors	Anti-HDAC (entinostat) *plus* anti-PD1 (pembrolizumab)	II	Melanoma, NSCLC	(57)
	Anti-HDAC (entinostat) *plus* anti-PD1 (pembrolizumab) *plus* anti-DNMT (azacytidine)	II	NSCLC	(58)
	Anti-VEGFR (axitinib) *plus* anti-PD1 (pembrolizumab)	Ib	RCC	(59)
	Anti-BRAF (dabrafenib) *plus* anti-MAPK/ERK (trametinib) *plus* anti-PD1 (pembrolizumab)	I/II	Melanoma	(60)
	Anti-PD1 (pembrolizumab) *plus* tyrosine kinase inhibitor (pazopanib)	I/II	RCC	(61)
	Anti-PDL1 (unspecified) *plus* CDK4/6 inhibitor (abemaciclib)	Preclinical	Breast, colorectal	(62)
Checkpoint blockade and IDO	Anti-IDO (indoximod) *plus* anti-CTLA-4 (ipilimumab) *or* anti-PD1 (pembrolizumab)	II	Melanoma	(63)
	Anti-IDO (epacadostat) *plus* anti-PD1 (pembrolizumab)	I/II	Solid tumors	(64)
Checkpoint blockade and Adoptive-T-cell Therapy	Anti-PD1 (BMS-936558) *plus* scFv-anti-Her-2 CAR T-cells	Preclinical	Metastatic breast carcinoma, fibrosarcoma	(65)
Checkpoint blockade and cancer vaccines or oncolytic viruses	Anti-PD1 (pembrolizumab) *plus* T-VEC viral vaccine	Ib	Melanoma	(66)
	Anti-PD1 (RMP14, BioXCell) *plus* dendritic cell vaccine	Preclinical	Glioblastoma	(67)
	Anti-PD1 (nivolumab) *plus* viagenpumatucel-L allogenic tumor cell vaccine	Ib/II	NSCLC	(68)
	Anti-CTLA-4 (ipilimumab) *plus* Coxsackievirus A21	Ib	Melanoma	(69)
Checkpoint blockade and radio/chemotherapy	Anti-PDL1 (atezolizumab) *plus* carboplatin chemotherapy *plus* anti-VEGF (bevacizumab) *plus* paclitaxel chemotherapy	III	NSCLC, carcinoma	(70)
	Anti-PD1 (pembrolizumab) *plus* pemetrexed *plus* carboplatin *or* cisplatin	III	NSCLC	(71)
	Anti-PD1 (nivolumab) *plus* doxorubicin *or* cyclophosphamide *or* cisplatin	II	Breast	(72)
	Anti-PD1 (pembrolizumab) *plus* palliative radiotherapy	II	Gastroesophageal squamous cell carcinoma, adenocarcinoma	(73)
Checkpoint blockade and nanoparticles	Anti-PD1 (pembrolizumab) *plus* NCP@pyrolipid (delivers oxaliplatin chemotherapy *plus* photodynamic therapy)	Preclinical	Colorectal	(74)
	Anti-PD1 (pembrolizumab) *plus* ZnP@pyrolipid (photodynamic therapy)	Preclinical	Breast	(75)
	SYMPHONY: anti-PDL1 (unspecified) *plus* laser irradiation *plus* gold nanostars	Preclinical	Bladder	(76)

Recent preclinical studies and clinical trials of combination therapies employ immunotherapy coupled with a second immunotherapy modality, as well as chemotherapy or radiotherapy (13, 24). Notably, combined checkpoint blockades involving PD-1 or CTLA-4 demonstrated significantly enhanced efficacy against advanced-stage melanoma, relative to targeting each alone (22). Currently, at least 20 single-agent and 3 combination immunotherapy regimens have been approved by the FDA (77, 78). The latter are nivolumab + ipilimumab against melanoma, bevacizumab+interferon-alpha for renal cancer, and elotuzumab + dexamethasone+lenalidomide for multiple myeloma (77). This trend is set to continue with increasing emphasis on rationally designed combinations in personalized settings (14).

## Emerging targets and combination therapies

In the following, we outline emerging targets and possible combinations with checkpoint blockers.

### Second generation immunotherapy targets

Many recent reviews have highlighted emerging alternative checkpoint inhibitors as targets for future monotherapies and/or inclusion in combination therapies (12, 13, 15, 32, 79). Whilst CTLA-4 and PD-1 checkpoint inhibitors are the crux of current clinical focus in immunotherapy, other checkpoints with potentially greater potency are emerging and promise to broaden the therapeutic “toolkit” and improve patient benefit. However, it remains essential to maintain the delicate balance between suppressive and stimulatory checkpoint modulation, using techniques such as multiplex immunoassays (80). VISTA, LAG-3, TIGIT, and TIM-3 immunomodulatory pathways are now well established as novel “next-generation” therapeutic targets (Supplementary Figure 1) (12, 15, 81–86). Most recently, P-selectin glycoprotein ligand-1 (PSGL-1), a glycoprotein with a critical role in cell adhesion and inflammation and regulator of T-cell responses in TME, was also found to be a potential “checkpoint” (87). Notably, ligating PSGL-1 to exhausted CD8+ T-cells inhibited T-cell receptor (TCR) signaling, decreased pro-inflammatory IL-2 and elevated PD-1 levels. Thus, PSGL-1 deficiency would reduce PD-1 expression and significantly enhance antitumor T-cell responses to melanoma (87). Anderson et al. postulated (i) that CTLA-4 and PD-1 could serve as “first-tier” co-target receptors responsible primarily for maintaining overall immune self-tolerance and (ii) that “second tier” receptors (TIGIT, LAG-3, and TIM-3), which have overlapping effects on NK and CD8+ T-cell effector functions, would exert more specific roles (85). LAG-3, TIM-3, and TIGIT are all highly expressed in dysfunctional T-cells in tumors. Synergizing their corresponding blockades would abrogate Treg cell-mediated immunosuppressive effects and enhance CD8+ and NK cell function within tumor tissues, demonstrating improved safety profiles over CTLA-4 and PD-1 inhibitors. Thus, emerging synergies of first- and second-tier blockades promise to produce stronger responses against a range of malignancies (85).

### Dual T-Cell checkpoint blockade

Rationale for synergizing anti-PD-1 and anti-CTLA-4 inhibitory mAb's is strong since both are expressed on T-cells but employ distinct, complementary mechanisms of action for suppressing T-cell function (Figures 4A–D) (12). A phase I trial of nivolumab (anti-PD-1) and ipilimumab (anti-CTLA-4) combination on patients with advanced melanoma produced an unprecedented 53% objective response rate (ORR), leading to two-year overall survival (OS) in 79% of cases (NCT01927419) (43). Later studies corroborated the findings in showing the superiority of the combination (ORR = 61%) compared with ipilimumab alone (11%); 22% of patients showed total remission (22). In phase III trials, anti-PD-1 alone, and anti-PD-1+anti-CTLA-4 combination were less toxic and had higher efficacy than anti-CTLA-4 alone. However, more cases of irAE were reported in the combination group relative to ipilimumab or nivolumab monotherapies (55 vs. 27% and 16%, respectively) (44). These included severe acute tubulointerstitial nephritis and a systemic rash but could be reversed with immunosuppressants and no fatalities occurred (NCT01844505) (44).

Prostate cancer is thought to be immunologically “cold,” lacking in tumor infiltrating lymphocytes (TILs), thereby limiting the effectiveness of PD-1 blockade which favors high-TIL tumors (88). Recently, however, Sharma et al. treated prostate cancer patients with anti-CTLA-4 (ipilimumab) and observed both elevated levels of T-cells in tumors and increased expression of PD-1 and VISTA inhibitory checkpoints. Consequently, combinations of checkpoint inhibitors targeting CTLA-4, PD-1, and VISTA would appear promising (88).

### Costimulatory mAb's

This approach aims to generate synergies between checkpoint inhibitors and costimulatory receptor mAb's (Figure 5). The first signal necessary for T-cell activation is triggered when APCs present antigens to TCRs via MHCs. The second/final signaling occurs when co-stimulatory receptors on T-cells (e.g., CD28) interact with compatible APC surface proteins (Figure 3). Progress in this approach was initially slow, owing to the clinical failure of the CD28 super-agonist mAb TGN1412 that induced “cytokine storms” and life-threatening organ failure in 17% of patients (89). More recent, promising trials incorporate agonist mAb's targeting costimulatory receptors including 4-1BB, GITR and OX40, that promote proliferation and survival of T-cells (12). Other “receptors” include CD27 (involved in long-term immunological memory of T-, B- and NK-cells) and CD40 (mediating antigen-presenting cell activation) (14). A phase I trial on metastatic melanoma patients (*n* = 24) with tremelimumab (anti-PD-1) and CP-893,870 (a CD40-agonist mAb) led to 27% ORR, 26-month OS and complete response in 8% of cases. Although 79% of patients developed cytokine release syndrome, this could be managed by standard care (NCT01103635) (51).

Co-stimulatory agonist mAb's targeting T-cell antigen 4-1BB are among the most advanced to be developed (15). This antigen is appealing given its expression on both T-cells and APCs, coupled with its ability to boost T-cell effector functions, expansion, and survival (90). In a murine colon adenocarcinoma model, significant synergy was reported for 4-1BB agonists plus PD-1 blockade combination resulting in total rejection of tumors (91). This effect involved increased levels of intra-tumor IFNγ-producing CD8+ and CD4+ T-cells, compared to monotherapies. Furthermore, the extent of irAEs was much improved and there was no overt toxicity (91). A further study on mice showed, however, that while 4-1BB mAb agonists alone halted progression of c-Myc-driven B-cell lymphoma in 70% of cases, combination of 4-1BB agonist with PD-1 blockade unexpectedly reduced this antitumor effect (92). Furthermore, concurrent PD-1 blockade and OX40 agonist in breast cancer murine models dampened the efficacy of OX40 agonist, with significantly reduced CD4^+^ and CD8^+^ lymphocyte infiltration, whilst applying OX40 agonist followed by PD-1 blockade enhanced efficacy, regressing breast tumors in 30% of cases (93). Consequently, simultaneous modulation of costimulatory and coinhibitory T-cell receptors warrants further investigation with careful consideration to the timing of the combination treatment (92).

### Checkpoint blockers with conventional therapies

Radiotherapy results in stimulation of DNA-damage repair mechanisms and release of proinflammatory cytokines and tumor antigens (12). Localized radiotherapy (even sub-therapeutic dosages) can also cause significant immunostimulatory regression of distant, non-irradiated tumors, known as an “abscopal effect.” The latter was exploited in a combination with checkpoint blockers (ipilimumab or pembrolizumab) against metastatic melanoma (94). Such coupling of checkpoint inhibitors with radiation significantly enhanced tumor CTL infiltration and elevated ORR in prostate cancer, NSCLC and glioblastoma (95). Furthermore, only low-moderate toxicity (~10% irAEs) was reported for combination of PD-1 or CTLA-4 blockade with radiotherapy against metastatic lung cancer (96). Interestingly, a triple combination of anti-CTLA-4 + anti-PD-1 + radiotherapy induced complete responses in mouse pancreatic cancer and melanoma models, not seen with dual-checkpoint blockade alone (13). In certain cases, however, radiotherapy + anti-CTLA-4 of patients with high tumor PD-L1 levels (type I TME) did not respond, contrary to anti-PD-1 treatment alone. Hence, future trials combining anti-PD-1 and radiotherapy could enhance ORR especially in patients possessing TMEs rich in PD-L1 expression and CD8+ lymphocyte infiltration (13, 24, 97).

Chemotherapy can also promote anti-tumor immune response by stimulating proinflammatory cytokines, reducing cytotoxic T-cell loss, and specific immunomodulatory effects (98). Examples of the latter include myeloid-derived suppresor cells (MDSCs) and Treg cell depletion by taxanes and cyclophosphamide, respectively (12). A phase Ib trial on advanced or metastatic NSCLC patients found that atezolizumab followed by carboplatin/nab-paclitaxel induced a response rate of 75% (*cf*. ~30% obtained with single-agent platinum doublet treatment) (NCT00527735) (99). More recently, a chemo-immunotherapy approach to murine colorectal cancer, combining oxaliplatin with PD-1 or CTLA-4 blockade proved synergistic by generating high levels of TILs and pro-inflammatory cytokines and downregulating inhibitory checkpoints (100).

A primary clinical objective is to convert “cold” non-immunogenic tumors into “hot” immunogenic tumors more receptive to immunotherapy by priming T-cells already present (101). In this regard, chemotherapy-based immunomodulation before checkpoint blockade shows promise. In a phase II trial, 50 metastatic triple negative breast cancer (TNBC) patients were given low-dose chemotherapy (over 2 weeks) followed by nivolumab. This produced ORRs of 24% and OS of 80% after 1 year, with an acceptable toxicity level, superior to existing anti-PD-1 monotherapies (NCT02499367) (72, 101). Thus, such “one-two-punch” strategies of low-dosage immunogenic chemotherapies plus checkpoint blockers (or cell cycle inhibitors or epigenetic modulators) can boost tumor immunogenicity. In turn, this promises to avert tumor relapse by destroying dormant cancer cells and/or enforcing their prolonged dormancy, and could be incorporated into future combination therapies (102). Indeed, a recent phase III trial, treatment of non-squamous metastatic NSCLC patients (n = 616) with pembrolizumab plus chemotherapy (cisplatin/carboplatin and pemetrexed) showed greatly improved efficacy relative to chemotherapy alone (ORR = 48 vs. 19%) with no change in the irAE level (NCT02578680) (71). Interestingly, OS was improved regardless of tumor PD-L1 expression levels, even in PD-L1 negative patients. This result would argue strongly for pembrolizumab + chemotherapy combination replacing chemotherapy alone as the standard of care for first-line treatment of metastatic NSCLC (71).

### Bifunctional agents

These include bispecific antibodies (bsAb's) and double-headed fusion proteins. bsAb's have dual specificity, binding simultaneously to two antigens, and high affinity (103). Bispecific T-cell engagers (BiTE's) represent an innovative format comprising two single-chain variable fragments (scFv's) joined in tandem via a flexible linker, where one antibody is specific for CD3 (a surface co-receptor on T-cells) and the other for a selected antigen on malignant target cells (104). Blinatumomab, the first FDA-approved bsAb/BiTE, binds T-cell CD3 and CD19-expressing B-cell acute lymphoblastic leukemia (B-ALL), thus eliminating tumors by redirecting T-cells onto them. The subsequent influx of granzyme proteases (derived from T-cells) enables a cytosolic synapse between T-cells and target cells, inducing apoptosis of B-cells (105); T-cells also proliferate and secrete pro-inflammatory cytokines including IL-2, TNFα, and IFNγ (105). In a phase II trial against refractory B-ALL, 43% of patients showed complete responses and a median OS of 6.1 months (NCT01466179) (106, 107). Blinatumomab and other BiTE antibodies aim to overcome tumor immune evasion mechanisms by directly engaging endogenous T-cells (104, 105). This could prevent the need (i) to expand and reintroduce T-cells, including specific clones, *ex vivo*, and (ii) to use costimulatory molecules (104, 105). Significant advantages over standard mAb's include enhanced cytotoxic potential, ability to bind weakly-expressed tumor antigens, superior protein stability and high potency in redirecting T-cells to target tumors even at low dosages (10–100 pg/ml) (104, 105, 108).

Cancer stem cells (CSCs) play a significant role in tumor initiation and progression, and their eradication is critical for preventing chemoresistance and eventual disease recurrence (109). The single-chain BiTE Solitomab (MT110) simultaneously targets the epithelial cell adhesion molecule (EpCAM) CD326, a transmembrane glycoprotein and promising CSC biomarker, and CD3 on T-cells (109). In a mouse model of human pancreatic CSCs, MT110 stabilized tumor growth and small remaining tumors contained no CSCs (109). BiTEs are being developed for a range of hematological and solid tumors, including ALL, non-Hodgkin lymphoma (NHL), glioblastoma, melanoma, and cancers of breast and prostate (109). However, some side effects of cytokine release syndrome (CRS) have been reported, and benefits appeared short-lasting possibly due to the small size of BiTEs (~55 kDa)/short half-lives, requiring repeated administering every 48 h (110). BiTE performance is also being evaluated in combination with anti-PD-1 + anti-CTLA-4 immune blockades to enable even greater T-cell activation (103).

Bispecific fusion proteins (created by joining parts of two different genes) are being used to simultaneously to block PD-1/PD-L1 and growth factor/cytokine signaling. A first such protein (M7824) has recently been investigated in phase I trials against several types of advanced solid tumors and has produced promising complete or partial response rates of up to 21% (NCT02517398, ongoing) (111). M7824 simultaneously blocks PD-L1 and TGF-β immune-inhibitory pathways to both restore and enhance host immune responses. The rationale is based upon averting the immunosuppression of effector T-cell function by PD-L1 and sequestering TGF-β (secreted by malignant cells, MDSCs, and Treg cells), hence preventing TGF-β-mediated tumor development and metastasis (111).

### Epigenetic modulators

Here, a checkpoint inhibitor is combined with an epigenetic modulator, such as an inhibitor of histone deacetylases (HDAC) or DNA methyltransferase (DNMT). This is viable since HDAC is commonly overexpressed in tumors and its inhibition downregulates the expansion of MDSCs that normally accompanies and promotes the cancer process (112). Additionally, most epigenetic drugs demonstrate only minor toxicity at clinical dosages (113). A major study focused on complementing the high-efficacy/short-term effects of targeted inhibitors with the low response rate/durable efficacies of single-agent immunotherapies (12). Mouse carcinoma models were used to examine the efficacy of ipilimumab (anti-CTLA-4) and nivolumab (anti-PD-1) synergized with 5-azacytidine (DNMT inhibitor) and entinostat (HDAC inhibitor). This eliminated >90% of colorectal carcinomas and 100% of metastatic mammary tumors. Rather disappointingly, however, phase II trials against NSCLCs reported durable, complete responses in only ~5% of patients (112, 114). 5-azacytidine also boosted antitumor immunity by dampening Treg cell function and upregulation of tumor cell antigen presentation machinery, critical to effective immune responses (115). Notably, demethylation of T-cell PD-1 promoters by 5-azacytidine in AML patients correlate with upregulated T-cell PD-1 expression (116). Consequently, DNMT inhibitor + PD-1 blocker combinations are being explored in ongoing clinical trials against NSCLC and other malignancies (115).

### Checkpoint blockers with targeted therapies

In this combination, checkpoint inhibitors are coupled with a modulator of growth factor signaling, mainly an inhibitor of protein kinase or phosphatase. In particular, receptor and non-receptor tyrosine kinases play a significant role in tumorigenesis as well as in immunogenicity and cytotoxicity (12, 117). Consequently, their inhibitors (TKIs) would offer natural synergy with checkpoint blockers. The angiogenesis-inducing growth factor, VEGF, restricts T-cell infiltration across the tumor endothelium and amplifies MDSCs and Treg cells within tumors. Against metastatic melanoma, combination of bevacizumab (a VEGF inhibitor) with ipilimumab induced a disease-control rate (DCR) of 67% and promoted T-lymphocyte infiltration of tumors with favorable tolerance. Combinations are now being sought that might synergize anti-PD-1/PD-L1 mAb's with VEGF blockade for even greater efficacy (22, 79).

In a mouse model of gastrointestinal stromal tumor (GIST), imatinib (a broad-spectrum TKI) was combined with an anti-CTLA-4 mAb to block T-cell immunosuppression mediated by indoleamine 2,3-dioxygenase (IDO). Synergistic activity was reported that reduced Treg cell population and enhanced tumor infiltration by CD8+ T-cells. Thus, CTLA-4 and IDO blockade combination significantly decreased tumor volume by 50% after 80 days, while during CTLA-4 blockade and imatinib administration alone tumors expanded by 40–60% over a similar period (117).

Ser/thr kinase and proto-oncogene BRAF promotes cell proliferation. In BRAF^V600E^ mutant melanomas, the specific inhibitor vemurafenib elevated MHCI induction and blocked immunosuppressive cytokine secretion (118). Unfortunately, however, a phase I vemurafenib+ipilimumab trial was canceled after significant level of irAE was reported (119). Later studies employed an extended combination (a BRAF inhibitor + a PD-L1 blocker + a MAPK/ERK inhibitor) to yield vastly improved efficacy (79, 120). Furthermore, numerous cancers express IDO that represses anti-tumor responses by depleting tryptophan—critical for T-cell effector activity and survival—and produce immunosuppressive metabolites. Accordingly, therapeutic interventions with PD-1 and IDO blockades have generated significant clinical potency and sustained objective responses. In phase II trials against advanced melanoma, a synergistic combination of the IDO inhibitor indoximod with pembrolizumab produced complete or partial responses in 52% of patients, with negligible toxicity (NCT01866319) (63). Relative to PD-1, a superior safety profile was reported with CTLA-4 blockade. Another IDO inhibitor, epacadostat, was recently combined with pembrolizumab against metastatic melanoma and demonstrated 74% DCR and 53% ORR, with only 5% experiencing irAEs (NCT02178722) (64).

Macrophage phosphoinositide 3-kinase-γ (PI3Kγ) serves as a critical regulator of immune suppression, supporting immunosuppressive myeloid cells within TME (121). In preclinical trials, inhibition of PI3Kγ with IPI-549 resulted in reprogramming of immunosuppressive macrophages (M2) into a pro-inflammatory (M1) state. This enhanced both activation and recruitment of cytotoxic T-cells to melanomas. Mice with melanoma rich in macrophages had dramatically enhanced response to immune checkpoint inhibitors and survival when co-treated with IPI-549: Monotherapy with anti-CTLA-4 or anti-PD-1 produced total remission in 20% of cases whilst addition of IPI-549 increased this to 80%. We should note, again, however, that IPI-549 would show benefit only against tumors with high myeloid cell content, so appropriate preselection of patients would seem essential for the best possible outcome of the co-treatment (122).

Colony-stimulating factor 1 (CSF1/M-CSF) also contributes to resistance of melanoma to PD-1 blockade (123). Activated CD8+ T-cells, upon releasing IFN-γ and TNF-α into the TME, experience a “vicious cycle” whereby these immunosuppressive cytokines trigger melanoma to adaptively secrete CSF1 (123). In turn, CSF1 promotes the differentiation and accumulation of pro-tumoral/inflammatory TAMs and MDSCs. In murine melanoma models driven by BRAF^V600E^, anti-CSF1 inhibitors alone displayed modest efficacy, yet dual blockade of anti-CSF1 and anti-PD-1 regressed 100% of tumors by 17 days, with 90% survival after 90 days (123). CSF1 inhibitors also showed additive effects with ACT therapies in preclinical melanoma studies, where anti-CSF1 dampened myeloid cell-mediated immunosuppression in the TME, permitting greater CD4+ and CD8+ T-cell infiltration into tumors and with improved functionality (124). Such effects could be beneficial to melanoma patients refractory to existing checkpoint blockade and ACT monotherapies.

IKKβ (IkB-Kinase β) represents a major component of the NF-kB signaling pathway, responsible for mediating T-cell development and activation (125). Mature Treg cells avert autoimmunity yet limit antitumor immune responses via CTL inhibition, and are heavily reliant upon NF-kB signaling for their development. Consequently, in melanoma murine models, IKKβ inhibition with KINK-1 (Kinase Inhibitor of NF-kB-1) reduced circulating Treg cells by ~50% with no change in CTL levels (125). The latter is due to CTLs being less reliant upon IKKβ for proliferation and survival than Treg cells. Thus, combining Treg-nullifying IKKβ inhibitors with other immunoactive pharmacological agents could bolster therapeutic efficacy (125).

Cyclin-dependent kinases 4 and 6 (CDK4/6) are core cell-cycle components, essential to initiation and development of breast cancer and T-ALL. CDK4/6 inhibitors (CDK4/6i) showed effectiveness against glioblastoma, breast cancer and melanoma by arresting tumor cell cycle at G1, via inhibition of retinoblastoma tumor suppressor phosphorylation (126). CDK4/6i can also induce antitumor immunity by overcoming two tumor immunoevasion mechanisms via (i) presenting tumor surface antigens with enhanced efficiency and (ii) inhibiting immunosuppressive Treg cell proliferation (62). Indeed, in murine breast cancer models, abemaciclib (CDK4/6i) + anti-PDL1 reduced tumor volume by 70% after ~2 weeks (stable up to 35 days) while abemaciclib or anti-PDL1 monotherapy was effective only temporarily. In colorectal CT-26 mice models also, this combination produced prolonged 100% regression, accompanied by resistance to further disease inducation (62).

### Checkpoint blockers with cancer vaccines

Sipuleucel-T (the only FDA-approved cancer vaccine) monotherapy has limited efficacy, probably due to T-cell inactivation resulting from TME-induced immunosuppression (12). In contrast, combination with checkpoint inhibitors appears promising, and Sipuleucel-T+ ipilimumab is currently in phase II trials against chemotherapy-naive prostate cancer (127). No benefit was reported from addition of nivomulab to a multipeptide vaccine against melanoma (12). On the other hand, poly(lactide-co-glycolide) vaccines combined with anti-CTLA-4 checkpoint inhibitors demonstrated promising results, regressing B16 melanoma tumors in mice and increasing survival rates by 75% (128). Another preclinical study showed that whole-tumor vaccines producing granulocyte macrophage colony-stimulating factor (GM-CSF) greatly enhanced anti-CTLA-4 blockade efficacy against pancreatic and prostate cancers. Compared with monotherapy, ipilimumab+sagramostim (recombinant GM-CSF) improved OS by 38% and reduced toxicity (37). Furthermore, whilst checkpoint inhibitor monotherapies proved ineffective against pancreatic ductal adenocarcinoma (PDAC) in mice, anti-PD1 blockade coupled with GVAX vaccine (secreting GM-CSF) improved survival, bolstered CD8^+^ effector T-cell production and enhanced T-cell PDA-specific IFNγ secretion within TME (129).

Significant progress has also been made against glioblastoma, commonly associated with extremely poor prognosis with <2 year median survival when treated with conventional chemotherapy (67). In contrast, PD-1/PD-L1 blockade following dendritic cell (DC) vaccination in a mouse model doubled the survival period (67). The fundamental mechanisms underlying this synergy were revealed as follows: (i) Glioblastoma requires substantial CD8+ NK cell infiltration; (ii) DC vaccination makes brain tumors permissive to T-cell infiltration; and (iii) subsequent PD-1 blockade neutralizes the suppressive checkpoint “shield” that would render T-cells dysfunctional (67).

### Checkpoint blockers with ACT

Adoptive-T-cell therapy aims to stimulate durable anti-tumor immune activity via (i) manipulation of T-cells *ex vivo*—TIL selection and expansion from patients before reinfusion—and (ii) gene therapy via sTCR (synthetic T-cell receptor) or CAR (chimeric antigen receptor) transfer into T-cells (130, 131). Checkpoint blockade prevents T-cell inhibition, as required by adoptive-T-cells for maximum anti-tumor activity, whilst its efficacy relies upon tumor-specific adoptive T-cells. So far, promising results have been obtained with CD19-specific CAR T-cell therapies, most notably against ALL, producing 90% total remission of which 67% showed sustained response after 6 months (132). High efficacy was also reported against advanced B-cell lymphoma (up to 53% complete response) (133).

The first FDA-approved CAR-T therapy Kymriah^TM^ (Tisagenlecleucel, CTL019) displayed durable remission in 83% of pediatric and young adult B-ALL patients tested (134). Also, CAR-T therapeutic Yescarta^TM^ (axicabtagene ciloleucel, CT019) won FDA-approval following the success against refractory aggressive NHL with 82% ORR, 54% CR, and 80% survival after 6 months. Neutropenia and leukopenia were the most frequently observed IrAEs but could readily be managed (135, 136). Furthermore, an ongoing trial progressing into phase II, tested anti-BCMA (B-cell maturation antigen) CAR-T therapy in late-stage relapsed/refractory multiple myeloma patients (NCT02658929) (137). Impressive 94% ORR, and 56% CR rates were reported. Importantly, 90% of patients were given “minimal residual disease” (MRD) negative status, indicating an absence of even very small residual malignant cells, often remaining during or after remission, and responsible for relapse (137). Consequently, such studies suggest potential for CAR-T therapies to move beyond lymphoma and leukemia, and toward other pressing hematological cancers such as multiple myeloma (137). In contrast, unfortunately, ACT therapies have proven ineffective against solid tumors, often inducing life-threatening side effects such as CRS and respiratory distress (138). Nevertheless, preclinical tests using transgenic Her2 murine models of breast cancer have given promising results: Addition of a PD-1 inhibitor boosted Her2-specific CAR T-cells function and proliferation, with enhanced regression, compared to CAR T-cell administration alone (65). A major priority for this type of combination, therefore, is to identify ideal, tumor-specific antigens as novel co-targets.

Efficacy of ACT for solid tumors is also marred by challenges in T-cell delivery. CAR-T cells only demonstrate optimal performance if the local environment is sufficiently nutrient-rich and waste products are easily removable (138). Accordingly, Smith et al. laced biopolymer scaffolds with the immune-stimulatory STING (stimulator of interferon genes) agonist cdGMP (cyclic di-guanosine monophosphate) and CAR-T cells (138). In addition to their primary function as a T-cell delivery vehicle, These bioscaffold implants release cd-GMP to convert tumors into self-renewing vaccine sites whereby destroyed tumor cells provide an antigen source that then launch a broader, second-wave antitumor response after CAR-T cell release (138). Thus, compared with conventional systemic T-cell injections, significantly improved tumor regression, without significant toxicity, was obtained in mice against both inoperable pancreatic cancer and melanoma (138). Future trials involving biopolymer platform may incorporate, in addition to CAR-T cells, checkpoint blockers and IDO inhibitors (138).

CAR-T cells were recently succesfully generated in the mouse bloodstream *in vivo* in large quantities and with high efficiency (139, 140). Nanoparticles (NPs) possessing surface anti-CD3e F(ab')2 fragments and microtubule-associated and nuclear localization sequences were intravenously delivered, enabling NP cargo delivery to leukemia-specific T-cell nuclei (139). NPs contained a DNA plasmid encoding a CAR-construct flanked by transposon elements, which was incorporated into the T-cell nuclei genome via a piggyBac transposase-mediated cut-and-paste mechanism (139). This technique circumvented the need for the expensive, overly elaborate and time-consuming *ex vivo* manipulation of CAR-T cells required by current CAR-T generation approaches (139, 140). Consequently, combinations of nanocarriers + CAR-T cells, perhaps synergized with checkpoint blockade, could enable efficacious clinical translation of CAR-T therapies in the future.

### Checkpoint blockers with nanoparticles

In a more recent approach, immunogenic “nano-scale coordination polymer” (NCP) particles, composed of oxaliplatin prodrug cores enclosed by a photosensitizer pyrolipid surface, were used to deliver chemotherapy and photodynamic therapy (PDT), respectively, to colorectal cancer in combination with anti-PD-L1 checkpoint inhibitors (74). NCPs represent a novel class of multimodality delivering self-assembled nanomaterials with a flexible composition that are biodegradable in host tissues. The “NCP@pyrolipid” hybrid nanostructure combines oxygen, light, and photosensitizers to generate unstable reactive ^1^O_2_ species that can destroy target tumors by promoting apoptosis and acute inflammation. Oxaliplatin was shown previously to induce immunogenic cell death (ICD) against colorectal cancer (74). Thus, a three-way synergy with (i) pyrolipid-induced PDT, (ii) oxaliplatin chemotherapy, and (iii) checkpoint inhibition was reported. In murine colorectal cancer models treated with an anti-PD-L1 (pembrolizumab) and NCP@pyrolipid combination, CD8+ T-cell densities in tumors increased by up to 10-fold (74). Furthermore, addition of localized PDT induced an abscopal effect: targeted tumors shrunk by 67% while distant tumors regressed almost completely. This “triple combination” may also be applicable to other metastatic cancers with PDT-accessible primary tumors (74). This was indeed confirmed in murine models for a combination of anti-PD-L1 (pembrolizumab) + ZnP@pyro + PDT against both primary and metastatic breast cancer (75). Nanoparticles that spatio-temporally delivered anti-PD-1 checkpoint inhibitors and agonistic anti-OX40 antibodies simultaneously to mouse 4T1 breast cancer significantly elevated T-cell stimulation via enhanced release of IFN-γ and increased CD8+:Treg cell ratio, resulting in doubling of survival rates (141).

On the other hand, development of immunomodulatory nanoparticle-based vaccines has been constrained by the tendency of phagocytes to sequester nanoparticles, blocking their access to target and leading to harmful accumulation in spleen and liver (142). Luo et al. have reported a versatile nanovaccine platform, in which a synthetic polymeric nanoparticle PC7A enhances cross-presentation of antigens, transports antigens to lymph nodes and functions as an immunogenic, tumor-suppressive adjuvant by activating “Stimulator of Interferon Genes” (STING) pathways (142). Consequently, upon ingesting PC7A, phagocytes are reprogrammed from “foe to friend.” The nanovaccine inhibited colon and melanoma tumor proliferation in preclinical mouse models (143). In the mouse TC-1 tumor model, synergy with PD-L1 blockade produced 100% survival even after 60 days, implying robust anti-tumor memory (142, 143).

Some success with SYMPHONY (“Synergistic Immuno Photodermal Nanotherapy”), a novel combination of gold nanostars (GNS), laser light and PDL1 blockade has been reported (76). Gold nanostars preferentially accumulate inside tumor cells due to their sharp spiked geometry, functioning as “lightning rods” that efficiently capture and convert laser light energy into heat, triggering thermic death of tumor cells deep within malignant tissues (76). Thus, SYMPHONY demonstrated significant superiority over anti-PDL1 monotherapy for both primary and distant metastatic bladder tumors (76).

### Oncolytic viruses

Oncolytic viruses aim to specifically infect tumors, then replicate within and destroy them, sparing healthy cells (144). However, whilst such therapy can also induce immune-based anti-tumor responses, host immunity can limit the spread and replication of OVs (144). Since checkpoint blockade is most effective in patients harboring immunogenic tumors, OVs are ideal combination candidates given their ability to induce TME immunogenicity by developing optimal conditions for T-cell priming and activation (145). Additionally, OVs have favorable safety profiles, with only mild flu-like symptoms reported (144). A modified herpes simplex virus taliogene laherparepvec (T-VEC) was FDA-approved after showing durable responses in 16% of resectable melanoma patients in phase III trials (146). More recently, CTLA-4 blockade was combined with CAVTAK^TM^, an immunotherapeutic and oncolytic strain of Coxsackievirus A21 (CVA21), itself an unmodified common cold RNA virus (147). In phase Ib trials on advanced melanoma, impressive 60% response rates and 78% disease control rates were obtained with only 8% irAEs (NCT02307149, ongoing) (69). In mouse models of aggressive ovarian and colon cancers, combination of OV expressing CXCL-11 (C-X-C motif of chemokine 11 precursor) with PD-L1 blockade markedly increased PD-L1 expression in TMEs, eliminated MDSC, Treg, and TAM immunosuppressive cells, and boosted T-cell infiltration, leading to complete responses in 40% of cases (148).

A pressing issue facing immune-oncology is converting immunologically “cold” and unresponsive tumors into more therapeutically receptive “hot” tumors, characterized by high CD8^+^ TIL infiltrate, PD-L1 expression and mutational load (149). Ribas et al. reported a promising phase Ib trial, where intralesional combination of talimogene laherparepvec (T-VEC) with PD-1 blockade induced 62% response rates (33% complete) in patients with advanced metastatic melanoma (66). Further work is needed to understand the mechanistic basis of this synergy (66).

As regards brain tumors, traditionally, OVs are injected intralesionally because of the blood-brain-barrier (150). However, a naturally-occurring human oncolytic *Orthoreovirus* was intravenously administered to patients prior to having their glioma tumors surgically removed within days. Resected tumors were then examined and were found to contain low quantities of viral capsid proteins in 100% of patients, indicating successful reovirus penetration into the brain (150). Minor side-effects of low-grade lymphopenia and minor flu-like symptoms were reported. Tumor PD-L1 expression is a major determinant of PD-1 inhibitor efficacy, and occurs at relatively low levels in glioblastomas (150). Critically, PD-L1 expression was significantly elevated in tumor extracts from reovirus-treated patients compared to controls, providing a rationale for synergistic combination with PD-1 blockade (151, 152). In a subsequent combination study on a immunocompetent orthotopic murine glioma model, sequential administration of reovirus for 2 weeks followed by PD-1 inhibitors for 1 week showed 100% survival up to 48 days, compared with monotherapies (36 days for reovirus and 22 days for PD-1 blockade (150). Furthermore, reovirus bolstered TIL recruitment, with 20% of total tumor cells containing CD8^+^ TILs in reovirus-treated mice vs. 2% in controls (150). Similarly, Maraba virus + PD-1 blockade in a murine model of breast cancer showed improved TIL infiltration, PD-L1 upregulation and survival relative to monotherapies (153).

Most recently, the Zika virus (ZIKV), well known to cause microcephaly and miscarriages in pregnant women across South America, has demonstrated significant oncolytic activity against glioblastoma in murine models (154). This was made possible by the ability of ZIKV to penetrate the blood-brain-barrier. Thus, Zhu et al. showed that mice treated with mouse-adapted ZIKV had significantly improved life-span (50% survival after 63 days vs. no survival after 30 days in the control group), with negligible effect on healthy neurones (154). Genetically engineered ZIKV strains could further improve safety, and future trials in combination with checkpoint blockade would seem promising (154).

### Synthetic biology: “gene circuits”

Major limitations of cancer immunotherapies include scarcity of tumor-specific antigens, direct immunosuppression by tumors and “off-target” toxicity (155). Recently, an immunomodulatory synthetic RNA-based “circuit” (two artificial tumor-specific promoters integrated with an RNA-based AND gate mechanism) was delivered by lentivirus into ovarian tumor cells to overcome such issues. Upon activation of both promoters exclusively within specific cancer cells, the AND gate expressed GAD fusion proteins to drive co-expression of a suite of immunomodulators termed “SCIP” comprising: (i) surface T-cell engagers (STE) and chemokines (CCL21) to label tumors for T-cell mediated destruction; (ii) IL12 cytokines to boost T-cell activation and effector activity; and (iii) PD-1 checkpoint blockade (155). In human ovarian cancer murine models, this approach resulted in stable SCIP-producing gene circuits, leading to unprecedented reduction in tumors to untraceable levels, and an OS level of 80 vs. 0% for controls. This effect was robust even when only 15–30% of the target tumor cells expressed SCIP. Such “gene circuits” can be expected to be applied to diverse malignancies (155).

## Potential, novel immunotherapy co-targets

In this section, we highlight a number of less widely recognized, emerging mechanisms that could potentially serve as co-targets in combination immunotherapy.

### Metabolic components

A number of metabolic mechanisms have been shown to be essential for immune evasion of tumors and could serve as co-targets in immunotherapy (15). Tumors demand an expansive, adaptable metabolic framework to thrive in specific niches, and all contemporary cancer hallmarks require metabolic engagement to some degree (156, 157). Recent evidence suggests that tumors may perpetuate their survival by reprogramming host metabolism (158). In patients with both anorexia and tumors, the increased metabolic stress causes elevation in systemic glucocorticoid hormones that alone can significantly decrease antitumor T-cell immune response, cause tumor growth and self-perpetuate the cycle (158). Novel combination approaches should therefore aim to normalize metabolic stress in parallel with checkpoint blockade to optimize clinical outcome. Notable metabolic targets of therapeutic interest include the tryptophan catabolizing enzyme indoleamine 2,3-dioxygenase (IDO), Notch homolog 1 (NOTCH1), and cyclooxygenase-2 (COX2) (159, 160–162).

Migration of immunosuppressive Treg cells to inflamed malignant tissues relies upon glucokinase-mediated glycolysis. Glycolysis is initiated by glucokinase (GCK), itself induced via the P13K-mTORC2 signaling pathway (163). Treg cells lacking components of this pathway remain immunosuppressive. Patients possessing a polymorphism causing elevated GCK activity saw enhanced Treg cell motility, given that GCK promotes cytoskeletal restructuring via actin association (163). Consequently, there exists potential for inhibition of glycolytic enzymes to manipulate the migration capacity of T-cell subsets, and thus to “soften” the immunosuppressive role of the TME (163).

Aerobic glycolysis, characteristic of growing tumors, fuels optimal T-cell effector function (159). In highly antigenic regressive tumors, competition for glucose in TME was found to be sufficient alone to drive cancer progression (159). This would occur as tumors surpass T-cells for glucose, directly sub-optimizing T-cell function by impeding their IFNγ production, critical for anti-tumor activity. Combination strategies that couple the depletion of tumorigenic immune cells with glycolysis enhancement in infiltrating T-cells, therefore, may prove effective at metabolic remodeling of the TME (159). This could also explain why combined anti-CTLA-4 + anti-PD-1 checkpoint blockade is particularly effective since anti-CTLA-4 would deplete Tregs whilst anti-PD-1 would directly dampen tumor glycolysis by inhibiting the mTOR pathway (159).

Drugs targeting tumor metabolism are in early trials. COX2 is essential for the production of the tumor-sustaining mediator prostaglandin E2 (PGE_2_), a prostanoid lipid that enhances cancer survival, metastasis, and immunosuppression (162). COX2 is overexpressed in several cancers, and COX2 inhibitors (e.g., aspirin) were found to act synergistically with PD-1 blockade in preclinical trials against melanoma, breast and gastric cancers (162, 164). COX2 inhibition would render melanoma and breast cancers vulnerable to immune control, restoring tumor immunosurveillance via CD8+ T and NK cell recruitment and promotion of the anti-tumor M1 macrophage phenotype (162, 164).

A recent metabolomics approach implicated CSCs in cancer metabolism. Chronic myeloid leukemia (CML) stem cells were found to be maintained by dipeptides accumulated via upregulated expression of Slc15A2 dipeptide transporters (165). *In vivo*, the internalized dipeptides would activate amino acid signaling via the p38MAPK-Smad3 pathway to sustain CML stem cell activity. Furthermore, TKIs could be synergized with the dipeptide transporter inhibitors. Such synergies showed highly specific responses in CML-affected mice where normal stem cells were insensitive to transport inhibitors (165). These observations would suggest that TKIs plus inhibitors of metabolic nutrient signaling within the p38MAPK-Smad3 pathway (e.g., Tocriset^TM^ and SIS3) could be beneficial to CML patients (Supplementary Figure 2) (165). Additionally, CSCs indirectly dampen antitumor immunity via secretion of the immunosuppressive factors TGF-β and arginase (to promote inhibitory Treg and TAMs) and attenuation of STAT-3 and PD-L1 surface proteins to inhibit antitumor CD8+ T-cells (166). Thus, a two-pronged therapeutic approach may be possible, where an initial TKI/anti-dipeptide transporter combination clears CSCs—a major component of tumor recurrence—and then an anti-PD-1 checkpoint inhibitor is added.

Metabolomics has also identified glutaminase as a potential target downstream of NOTCH1 (161). The latter is a conserved single-pass transmembrane receptor, crucial to T-cell lineage commitment and itself a major T-ALL target, given that activating NOTCH1 mutants are common in T-ALL (161). Glutaminolysis was identified as an integral pathway for leukemia cell proliferation controlled by NOTCH1 and, therefore, a critical determinant of anti-NOTCH1 clinical efficacy. Consequently, glutaminase + NOTCH1 inhibitor combinations showed a potent synergistic anti-leukemic effect *in vitro* in patient-derived T-ALL murine models (161) (Supplementary Figure 3).

In conclusion, metabolomics promises to identify novel therapeutic targets and mechanistic insights, and may provide important links reflecting the role of genetic, microbiome, lifestyle, and environmental factors in tumor development (167).

### Exosomes

**Figure 7:**
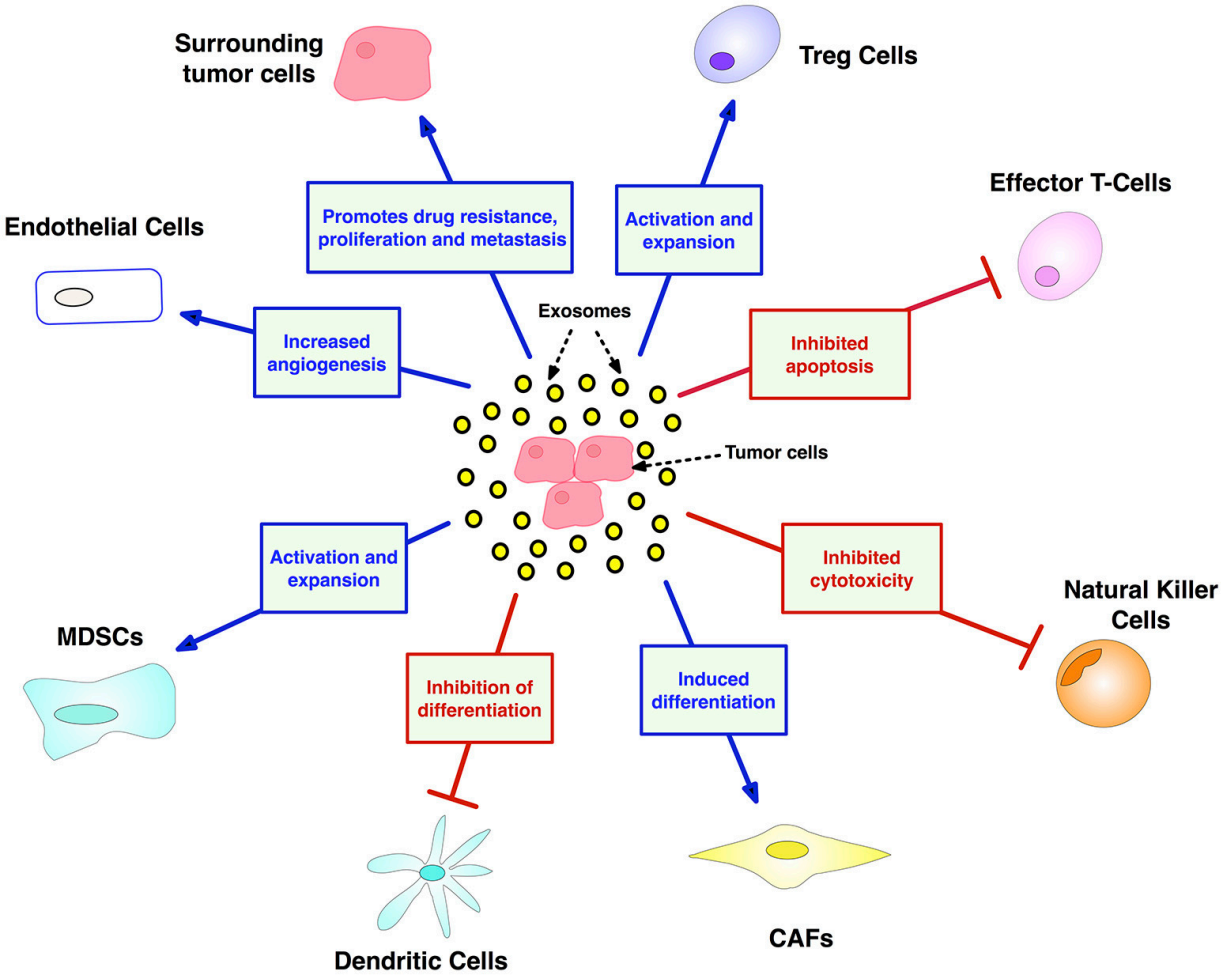
Exosome contributions to cancer facilitated by transport of oncogenic nucleic acids and proteins. Exosomes have major and diverse roles in tumorigenesis, including: (i) promoting an immunosuppressive TME by dampening NK and T-cells, while expanding inhibitory Treg and MDSC populations, (ii) mobilizing neutrophils, and thus skewing marcophages toward their M2 immunosuppressive form (iii) maintaining tumor drug resistance by exporting antitumor drugs and shuttling multi-drug-resistant proteins (iv) support tumor thrombosis and angiogenesis by activating endothelial cells (v) promoting metastasis by converting fibroblasts into myofibroblasts. CAF, cancer-associated fibroblasts; MDSC, myeloid-derived suppressor cell. Created using information from Zhang et al. (168).

Exosomes are specialized, nano-sized lipid bilayer vesicles that enable a novel means for intercellular communication, shuttling bioactive DNA, mRNA, miRNA, and oncogenic proteins between cells, thereby enabling genetic reprogramming of cellular networks (168). Various stages of the cancer process involve exosomal interactions (Figure 7). Thus, exosomes transmit messages from tumor cells to both stromal and immune cells, facilitating immune evasion, and establishment of the tumor niche (168). Exosomes may be therapeutically exploited via three approaches, as follows:

- *Direct exosome-based immunotherapy*. This is exemplified by “dexosomes” (dendritic cell-derived exosomes) loaded with whole antigen or peptide fragments, and have proven ability to induce systemic T-cell responses (169). Immunostimulatory dexosomes are especially promising, stimulating antitumor responses with greater accuracy than possible using non-cellular approaches, and possessing higher biostability and bioavailability as well as cost-effectiveness compared with other cellular therapies (169). Treatment of human breast cancer with dexosomes resulted in incorporation into tumors and subsequent expression of dexosome immunostimulatory molecules (e.g., CD86, CD81, MHCI/II + tumor antigen) on tumor cell surfaces, thus boosting tumor immunogenicity and T-cell engagement (170). Dexosome-treated tumors indeed contained a much higher proportion of T-cells secreting IFN-γ immunostimulatory cytokines (170). However, early clinical trials on colorectal and NSCLC have yielded only moderate efficacies (171). Efficacy may be improved by better composition/antigen-loading strategies and trafficking of exosomes (171, 172).
- *Exosome elimination in patients with advanced cancer*. This represents a new treatment concept, demonstrated for the blood-pressure dampening drug, amiloride. This decreased MDSC immunosuppressor functions in colorectal cancer patients by inhibiting exosome formation (168).
- *Exosomes as “natural nanoparticle” drug delivery vehicles*. As such, exosomes exhibit favorable biocompatibility and biodistribution (173). Indeed, use of macrophage-derived exosomes to transport paclitaxel into multidrug resistant (MDR) tumors enhanced treatment efficacy by 50-fold relative to paclitaxel administration without exosomes (173).

In conclusion, given their diverse roles in facilitating both tumorigenesis and immunosuppression, tumor-derived exosomes (TEXs) are attracting increasing interest as therapeutic targets (174). Recently, genetically modified K562 leukemia-derived exosomes expressing IL-15, IL-18, and 4-1BBL surface proteins displayed a biphasic effect upon human NK cells (i) enhancing cytoxicity and proliferation (4 h) and (ii) inhibiting activated receptor expression and dampening cytotoxicity (48 h) (175). These results raise the possibility of using TEXs as anti-tumor vaccines (175). More data is needed, however, to assess the clinical potential and safety of exosomes as mono- and/or combination-targets (168).

### Dividing T-Lymphocytes

The asymmetrical division of T-cells observed in murine models represents a novel opportunity as an unconventional immunotherapy target (176). When a “mother” T-lymphocyte naive to immune stimulation undergoes mitosis, mTORC1 (an enzyme responsible for protein synthesis) is divided unevenly between the two daughter cells. The progeny with the higher mTORC1 becomes strongly activated as a potent killer T-cell whilst the “sister” cell displays behavioral traits more associated with memory T-cells (176). This raises the possibility of exploiting mTORC1-expressing T-cells as a target for long-term potentiation of immunotherapy, by skewing development toward memory T-cells (176). Proteasome-activators such as Cyclosporine were found to tip the balance of dividing CD8^+^ T-cell progeny toward memory T-cells (exploiting the fact that effector and memory T-cells have differing proteasome activity levels) (177). Thus, there exists potential for synergizing immunotherapy with proteasome modulators (177).

### Ion channels

A variety of ion channels, including voltage- and ligand-gated ion channels, are expressed in cells of the immune system and make significant, dynamic contributions to immune functioning (178). Here, we highlight voltage-gated sodium channels (VGSCs/Nav's), voltage-gated potassium channels (VGPCs/Kv's), and calcium-activated potassium channels (K_Ca_'s).

VGSCs may manifest themselves in immuno-oncology and serve immunotherapy in several different ways. *First*, Nav1.5 was shown to control the positive selection of CD4+ T-cells from CD4+/CD8+ thymocytes in response to stimulation by APCs (179, 180). The selected cells would play a central role in immune functioning via production of cytokines and chemokines, facilitating antibody production by B-cells, maintaining immunological memory and priming CD8+ CTLs. Consequently, VGSC blockers could reduce the CD4+:CD8+ ratio, thus boosting CD8+ CTL populations that drive early immunosurveillance antitumor responses. Furthermore, high CD8+ TIL content of tumors is predictive of pathological complete response to primary systemic therapy regardless of cancer subtype (181). However, as a monotherapy, VGSC blockers may yield only short-term success since depleted CD4+ T-cells would ultimately reduce immunological memory and compromise CTL tumor re-challenge (182). Accordingly, tumor vaccine delivery after VGSC inhibition as a sequential “one-two punch” could activate new thymic CD4+ helper T-cells to restore lost immunological memory and sustain efficacious CTL antitumor responses (182). Additionally, VGSC blockers would increase tumor “hotness” by enhancing CTL presence, and thus synergize with PD-1 blockade (149). *Second*, functional VGSC expression occurs in macrophages, another cell type in the innate immune system. When recruited to tumors, macrophages can accelerate cancer progression. A recent study by Roh-Johnson et al. on zebrafish and mouse models of melanoma showed that recruited macrophages transferred their cytoplasm into melanoma cells and this promoted metastasis (183). How such intracellular communication is regulated and the nature of the transferred molecules were not known (183). Interestingly, however, VGSC activity drives macrophage motility (184). Accordingly, VGSC blockade could eliminate this component of immune response and could form the basis of mono- and/or combination immunotherapy with tumor vaccines or with PD-1 blockade to dampen TME immunosuppression, overcome PD-1 resistance and enhance patient responses (149). *Third*, the predominant VGSC in cancers of breast and colon is the neonatal splice variant of Nav1.5 (nNav1.5) (185, 186). This offers several advantages as a target, including having an extracellular region that can be targeted by an antibody and a restricted expression pattern in the adult human body (187, 188). Accordingly, nNav1.5 exprssion may be cancer-specific and could form the basis of CAR-T immunotherapy. Importantly, in all such cases, VGSC blockers would additionally suppress the invasiveness of the tumor cells themselves (189, 190).

The role of Kv1.3 channels in the immune responses to tumors may be more complex, dependant dynamically on disease stage (191). On the one hand, tumor infiltration may involve downregulation of the channel (192). On the other hand, Kv1.3 (and K_Ca_3.1) channels are expressed predominantly in CD8+ T-cells and contribute to membrane electrogenesis and calcium influx, crucial to their antitumor granzyme B and cytokine production (193). Kv1.3 activity also promotes T-cell proliferation and high-level expression of Kv1.3 correlated with elevated levels of Ki-67 (193). Finally, a novel novel role for Kv1.3 has been proposed in TME where cell death within a necrotic region can release cellular K^+^ into the extracellular spaces (194). Exposure of T-cells to such high K^+^ can suppress their activation and functioning by increasing intracellular K^+^ and inhibiting PP2A-dependent/TCR-activated Akt-mTOR signaling (194). Accordingly overexpression of Kv1.3 restored antitumor T-cell functionality by facilitating efflux of the high intracellular K^+^, leading to enhanced survival of tumor-bearing mice (194). Overall, therefore, Kv1.3 expression in T-cells can promote the immune reaction to tumors once the cells enter TME.

Calcium-activated potassium K_Ca_3.1 channels are upregulated in activated T-cells and also play a significant role in regulating cellular migration and proliferation (195). Upon activation by tumor cells, adherent NK (A-NK) cells preferentially up-regulated K_Ca_3.1 channels (196). Blocking K_Ca_3.1 activity with TRAM-34 increased the degranulation and cytotoxicity of A-NK cells, and induced increased ability of A-NK cells to reduce tumor growth *in vivo*. Taken together, these results rationalize the co-targeting of K_Ca_3.1 and PD-1 on NK cells in future cancer immunotherapy (197, 198). NK cells suppress metastasis by inducing degranulation-mediated tumor cell lysis via release of perforins and cytotoxic granzymes. K_Ca_3.1 blockers TRAM34 and NS6180 increased NK cell proliferation and enhanced degranulation rate of the non-adherent K562 erythroleukemia cells *in vitro* (199). On the other hand, Kv1.3 blockers Stichodactyla toxin (ShK) and 5-(4-phenooxybutoxy) psoralen (PAP1) decreased proliferation and degranulation, consistent with Kv1.3 being essential to NK-induced cytotoxicity (199).

**Figure 8:**
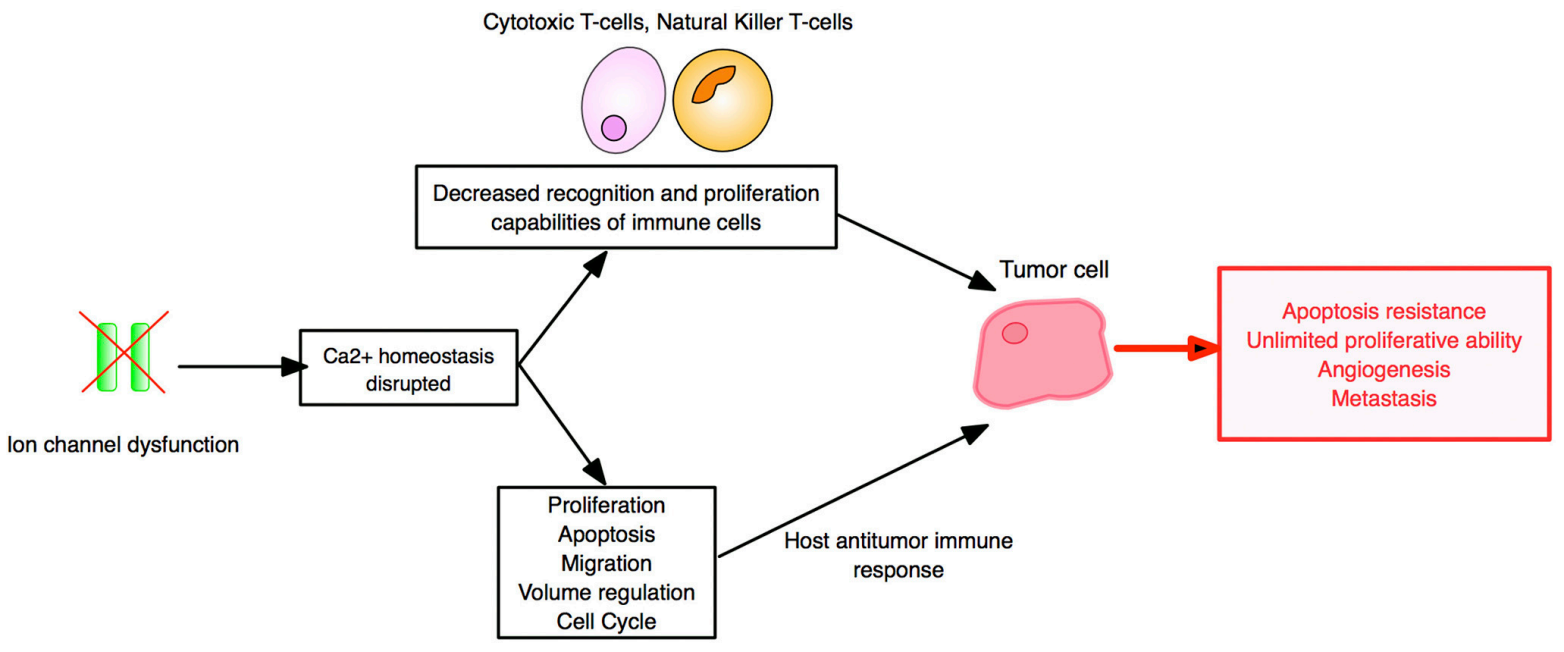
Ion channels as cancer immunotherapy targets. The impact of ion channel dysregulation upon tumor-immune system interactions is depicted. In both immune and tumor cells, ion channels are involved in regulating Ca^2+^ influx and downstream signaling pathways. Dysregulation of ion channels can directly fuel carcinogenesis, and immune cell cytotoxicity is dampened by alterations in Ca^2+^ signaling. Cancer hallmarks are boxed in red. Created using information from Bose et al. (200), Litan and Langhans (201), and Panyi et al. (193).

In conclusion, VGSC (in particular, nNav1.5), Kv1.3, K_Ca_3.1, and probably other ion channels and transporters represent novel immunotherapy targets. Importantly, since ion channels are also involved at all stages of the overall cancer process, their blockers may offer unique multi-faceted advantages for T-cell based immunotherapies, including combinations with PD-1 blockade (196, 198, 200) (Figure 8). A further advantage of ion channels is their ability to be manipulated remotely using optogenetics techniques (202).

## Personalization of immunotherapy

**Figure 9:**
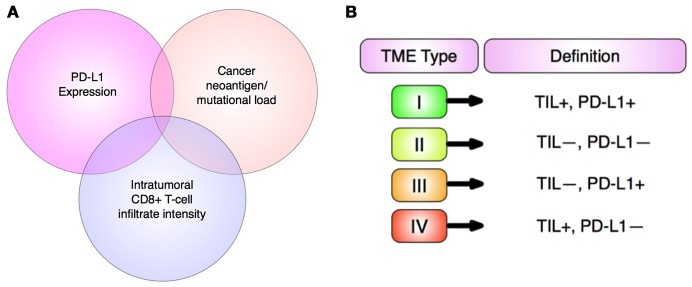
Personalized immunotherapy. **(A)** Multifactorial biomarker panels. The three most widely established biomarkers of response to anti-PD-L1 immunotherapies have strong functional overlap, hence all three will soon be used together to provide stronger predictive value of therapeutic outcome than single biomarkers. Adapted from Topalian et al. (203). **(B)** TME stratification into 4 categories based upon PD-L1 expression and presence of TILs (tumor infiltrating lymphocytes) by Teng et al. (204). This promises to enable prediction of patients that respond to checkpoint blockades, namely anti-PD-1 and anti-PD-L1 (204).

Checkpoint inhibitors can be applied to a wide spectrum of solid tumors and hematological cancers (132). This is possible given the conserved upregulation of PD-1 and other immune pathways across a plethora of different human cancers (203). However, PD-1 blocking drugs do not demonstrate efficacy against the full range of malignancies and not all patients with PD-L1^+^ tumors yield a response. This has been attributed to the phenomena of “mixed tumor regression” in which malignant growths at different locations within the body (even within a given tumor) of a patient display variable therapeutic responses (203). Thus, currently, only a limited subpopulation of patients can benefit (12). In order to enhance treatment efficacy, reliable biomarkers—ideally several screened simultaneously in multiplexed assays—are essential to identify patients likely to give the best response to a given immunotherapy regimen with minimal toxicity (203) (Figure 9A). CD8+ density, mutational load and PD-L1 expression as solitary biomarkers are not sufficient to effectively characterize the TME given their complex interdependency (205). Tumor mutational burden (TMB) indicates the total number of somatic mutations/Mb of DNA, where high TMB tumors (melanoma and NSCLC) are more likely than low TMB tumors (ovarian and breast carcinomas) to harbor foreign neoantigen proteins, resulting from these mutations (206). Checkpoint blockade can then stimulate and enable host immunity to detect neoantigens and destroy the tumor, demonstrating greater efficacies against higher TMB tumors (206). While TMB calculation is necessary, alone it is not indicative of checkpoint blockade response and therefore of limited clinical use (207). However, it could serve as a component of an integrated algorithm (also involving host genetics, microsatellite instability, neoantigen load, TIL content, the TME, and microbiome) to accurately determine patient response to immunotherapy (207, 208). Effective biomarker quantification is also essential for early detection, functional diagnosis and monitoring of treatment efficacy/disease progression (209). In these regards, novel biomarker-driven surrogate endpoints of PFS and ORR should be identified in order to earlier identify clinically meaningful responses (210–212).

Lesterhuis et al. proposed that effective therapeutic response to immune checkpoint blockade should follow a “critical state transition” comprising the following: (i) an initial stable state (where static, pre-treatment biomarkers are obtained at a single time point); (ii) a pre-transition state (following treatment initiation, from where the system can still revert back to the stable state); (iii) a critical transition state (reached when a biological “tipping point” or a small change to the system from checkpoint blockade occurs); and (iv) a new stable state (where malignant tissue reverts back to healthy tissue) (213). Emerging dynamic biomarkers, associated with treatment response, can be obtained from biopsies at multiple time intervals comparatively in non-responding and responding patients. Importantly, a “network biology” approach might identify dynamic biomarker “warning signals” near the “tipping point” by mapping molecular changes associated with tumor regression after checkpoint inhibitor therapy (213). This approach can potentially identify “gene hub products,” driving response, that could serve both as dynamic biomarkers and novel drug targets for combination therapy. Targeting such hubs can significantly increase both the proportion of patients benefiting from treatment as well as the overall magnitude of responses to checkpoint blockade (213).

Khalil et al. discussed the potential for personalized immunotherapy based upon tumor phenotype (15). Checkpoint immunomodulators are predicted to yield the best therapeutic response in tumors harboring a high neoantigen or mutational burden, immunosuppressive TME and high density of TILs, ideal for melanomas and lung adenocarcinomas (4, 15). CAR T-cell therapy would be optimal against leukemias and medulloblastomas that possess immune-permissive TMEs and lower antigenic diversity (15). This strategy can be used to design hybrid combinations of CAR T-cell therapy and checkpoint blockade that might address tumors with intermediate phenotypes, such as myelomas, and cancers of prostate, ovary, kidney, and liver. Heterogeneity of TMEs can also be characterized by single-cell flow cytometry analysis so as to evaluate possible inherent immune evasion mechanisms (214). Tumors can thus be stratified by phenotype to enable appropriate personalized treatment (Figure 9B) (5).

As already emphasized, the “immune contexture” (i.e., spatial organization, density and composition of the tumor immune infiltrate and function) directly influences cancer progression (215). For example, CD8+ T-cell density correlates with good prognoses in breast, colorectal and head and neck cancers, but poor for RCC. The influence of tertiary lymphoid structures is beneficial for pancreatic and breast cancer and NSCLC, yet negative for hepatocellular carcinoma. Treg cell abundance affects colorectal and gastric cancer prognoses positively, but is detrimental for breast, pancreatic cancers, and NSCLC (215). Decoding the immune (I), vascular (V), and stromal (S) components of TMEs via *in situ* immunophenotyping, immunohistochemistry and “omics” technologies revealed that checkpoint inhibitors perform optimally for tumors with high I, low V and low S content. CAR-T, vaccination and chemo- or radiotherapy are ideal for TMEs lacking any of these features, and combinations are suitable for malignancies with low I and high V and S (215). Thus, uncovering other predictive biomarkers for immune blockade responses—such as CTLA-4/PD-1 expression on immune infiltrate and tumor cells, TILs, and circulating MDSCs and lymphocytes—should be prioritized in order to improve the efficacy of personalized treatments of patient (215, 216).

A multiplex immunohistochemical imaging system (HALO^TM^) has been devised that enables simultaneous use of five different stains and permits analysis of any organelle or subcellular compartment (217). Such a system would be ideal for immuno-oncology where several biomarkers are needed to characterize distinct tumor and immune cell populations within tumor biopsies. Specifically, HALO^TM^ *Proximity* and *Tumor Infiltration* analyses allow precise quantitation of (i) the spatial relationship between the two cell populations and (ii) the position of immune cell density relative to the invasive tumor margin, respectively (217). In another technical approach, MultiOmyx multiplexed TIL panels yield comprehensive immunophenotypic profiling of tumors even from a single tissue section (218). Successful analyses of immune responses within solid TMEs have revealed two basic categories: high-TIL and low-TIL tumors (218). Such immunophenotypic analyses can facilitate personalized treatment regimens whereby high-TIL tumors would be treated effectively with checkpoint inhibitor monotherapies. In contrast, low-TIL tumors would respond most effectively to combination immunotherapies incorporating an agent that boosts the endogenous anti-tumor response (218).

**Figure 10:**
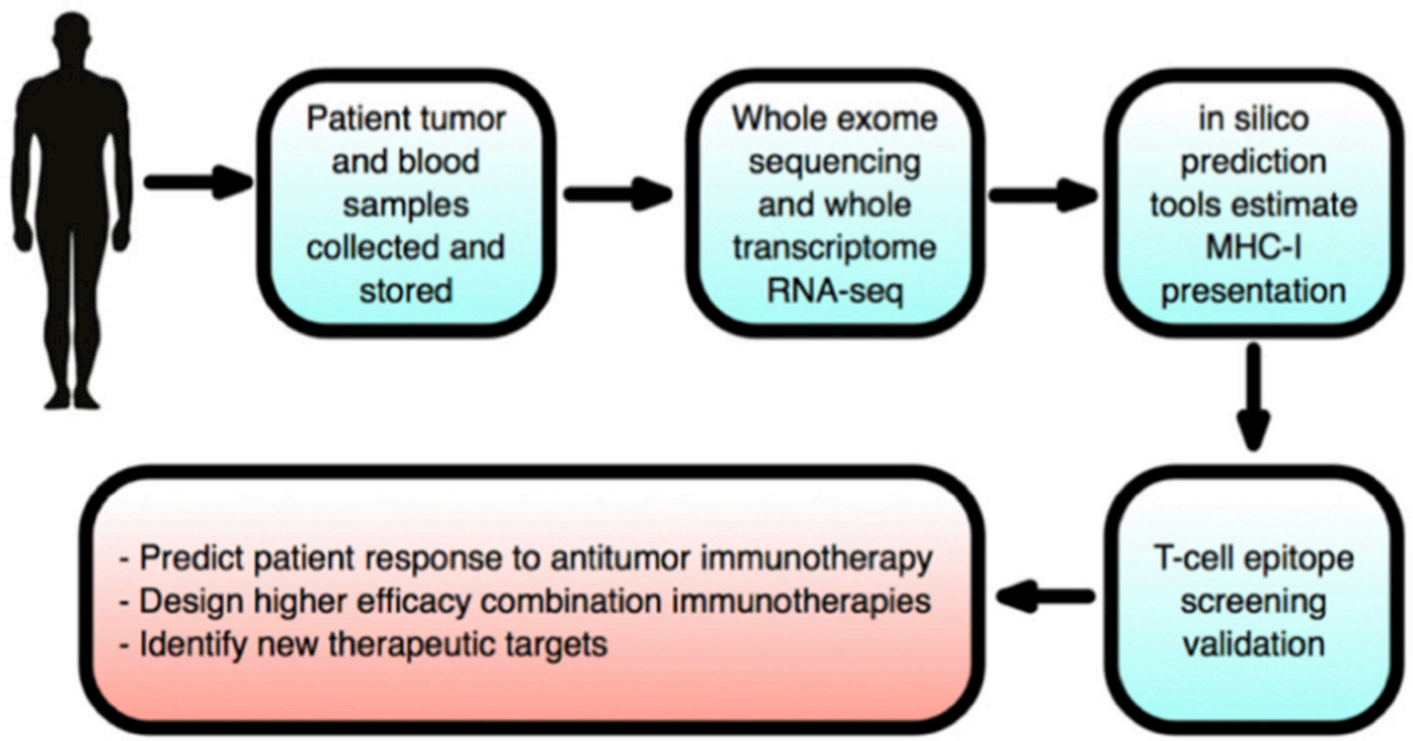
New neoantigen discovery pipeline to facilitate personalized immunotherapy. Neoantigens are mutated antigens that are unique to cancer cells, foreign to the immune system and severely limit immunotherapy efficacy. Checkpoint inhibitors stimulate and enable host immunity to detect neoantigens and destroy tumors. Following collection of patient tumor and blood samples, whole exosome sequencing on malignant and non-transformed cells derived from the same patient reveal tumor-specific mutations. Subsequently, whole-transcriptome RNA-sequencing establishes mutation expression levels, before *in silico* tools can identify neoepitopes (tumor-specific mutation-derived peptides presented on tumor cells via the MHC-I and recognized by T-lymphocytes). To deduce the most immunogenic subset of neoepitopes, and hence most promising of immunotherapy targets, T-cell assays are then run. Neoantigen discovery will therefore drive the design of neoantigen-specific vaccines and effective combination immunotherapies, and enable estimation of patient clinical response to treatments. Furthermore, neoantigen load estimation will therefore enable improved, personalized immunotherapies. Created using information from Schumacher and Schreiber (224), and Kvistborg et al. (225).

Expression and (co)localization of as many as 12 biomarkers can be quantitatively and qualitatively analyzed in single biopsy sections (218). Increasingly, genetic mutations in tumors are being profiled as the bases of patient suitability for specific therapies (219, 220). Furthermore, it is possible to profile DNA mutations in “liquid biopsies” in the form of ctDNA (circulating tumor DNA) shed from tumor cells into blood (221). Such procedures have the additional advantages of being relatively non-invasive and potential to detect therapy resistance-promoting mutations not readily revealed in tissue biopsies and to enable monitoring of tumor progression over time (221). Ultimately, liquid biopsies may complement tissue biopsies to refine immunotherapies and may not replace tissue biopsies at least in the short-term (222, 223). Tumor biopsies can also be subject to parallel (whole-exome and RNA-based) sequencing to identify tumor-specific mutations and associated neoantigens which would normally enable the immune system to differentiate between host and malignant cells (Figure 10). Neoantigens represent potential biomarkers of immunological activity and have been used to design personalized synthetic DNA, RNA, or peptide vaccines that target patient-specific neoantigen spectra. The most immunogenic mutant peptide epitopes from *in silico* prediction algorithms can then be introduced into the vaccine formulation and combined with PD-1 and/or CTLA-4 checkpoint blockade (Figure 10). Such personalized therapies can be engineered to enhance neoantigen-specific T-cell activities, and could also expand the cancer types that can be treated by immunotherapy.

Searching “The Cancer Genome Atlas” (TCGA) with algorithms, such as domainXplorer, identified 122 potential cancer driver genes corresponding to mutational status of TIL proteins (226). Furthermore, TCRs can be provided by neoantigen-sensitive donor T-lymphocytes, and can be wielded to retarget naïve patient T-lymphocytes and re-engage tumors (4). Clinical response durability could be improved by simultaneously targeting several neoantigens homogenously-expressed in tumors (4). Such tools could ultimately generate a comprehensive archive of drivers of gene mutations modulating anti-tumor immunity and thus enable design of specific immunotherapies.

Whole-genome sequencing of individuals with adult T-cell leukemia unveiled a novel genetic mechanism tied to immune evasion involving structural variants of the 3′ terminal of PD-L1 (227). Such variants, involving deletions, duplications, and other break points, would boost functional PD-L1 expression to abnormally high levels. Murine models with intact 3′ PD-L1 termini showed both enhancement of T-cell proliferation within the lymphoma TME and tumor regression (227). This would suggest that the 3′ PD-L1 terminal could serve as a diagnostic biomarker enabling selection of patients that would best respond to PD-L1 blockade therapies (227).

Importantly, as coding regions only account for <2% of the human genome, understanding the impact of the even higher numbers of non-coding somatic mutations on cancer is a priority (228). A systematic analysis of 930 tumor whole genomes and transcriptomes revealed that a network of 193 non-coding loci were disrupted in 88% of tumors tested, indicating widespread effects of non-coding mutations across diverse cancers (228). Notably, *in vitro* studies showed that a non-coding mutation upstream of DAAM1 upregulated its expression, enhancing the invasiveness of tumor cells. Whether or not different tumor subtypes have common patterns of both non-coding and coding mutations, and the intricacy of the global transcriptional network linking non-coding mutations to tumor gene expression, requires further investigation (228).

Bioinformatic analysis of 10,000 tumors across 33 diverse cancers by the Cancer Research Institute (CRI) iAtlas suggested the existence of six major immune sub-types (229). Notably, C2 (IFN-γ dominant) and C3 (inflammatory) subtypes were associated with good prognoses, while C4 (lymphocyte-depleted) and C6 (TGF-β dominant) had the most negative prognoses. C4 and C6 both possess high M2 macrophage and low TIL level signatures, corroborating earlier studies where such immunosuppressed TMEs were associated with poor outcomes (229). Given that the TME provides important insight into patient prognosis and treatment response, tumor immune sub-types could provide an invaluable role for predicting disease outcome (229).

Patient-derived xenografts (PDXs) in which human tumor fragments are implanted into immunosuppressed mice show superior recapitulation of tumor heterogeneity, preservation of gene expression profiles and response to conventional chemotherapy than conventional xenografts (230). Thus, PDXs can facilitate personalized immunotherapy (230). Limitations include time (2-12 months are needed to establish the models) and metastases being difficult to observe (230). Consequently, humanized swine “oncopig” models are in development that better mirror the human condition, including pharmacokinetics (231).

A non-invasive and cost-effective radiomics-based approach was used recently to convert subjective CT imaging into objective data and thus enabling “biomarkers” predictive of patient response to immunotherapy (232). A radiomic score was established based upon TIL quantity estimated from 6 parameters. Application of this technique to 137 CTs of neck, bladder, lung, and head cancers revealed that higher scoring patients had 50% greater chance of survival in PD-1/PD-L1 trials than patients with lower scores (232). Such quantitation of personalized approaches to immunotherapy is promising and should be expanded.

FDA recently approved the first pan-tumor biomarker as a test for microsatellite instability (MSI) in connection with pembrolizumab (Keytruda) (233). This can test all advanced tumor types for highly elevated MSI-H and/or its underlying trigger, the DNA mismatch repair (dMMR) system, and thus enable exclusive molecular indication for immunotherapy (233). Approximately 4% of all tumors possess the hypermutational MSI-dMMR phenotype, significant levels of neoantigens/high immunogenicity and these are the cancers most susceptible to checkpoint blockades (233). Indeed, promising results have been reported for MMR-deficient pancreatic or prostate cancer patients: 30% showed complete response to therapy, 23% showed partial responses, and 17% stabilized disease (233).

In conclusion, significant advances have been made in the personalization of immunotherapy. More work is required, however, to develop robust novel biomarkers (and their combination) to fulfill the potential of precision immunotherapy.

## Conclusions and future perspectives

Whilst overwhelming evidence supports the potential of immunotherapies in clinical oncology, much cross-disciplinary work remains to be done to maximize patient benefit. *First*, there remains strong demand for realistic preclinical models that can predict optimal combinations to minimize adverse side-effects and toxicities, and better interrogate immunotherapy mechanisms of action. Emerging 3D *in vitro* models are sharpening our understanding of tumor-immune cell interactions. The effect upon T-lymphocyte functioning of tumor associated fibroblasts (TAFs), a significant component of the TME and enhancer of metastasis, was evaluated in breast cancer using chitosan-alginate scaffolds (32, 234). This study concluded that TAF inhibition may boost the efficacy of adoptive cellular therapies and that such 3D scaffold assays could bridge the gap between *in vitro* experiments and preclinical animal models for evaluation of immunotherapies (234, 235). Probing the intricate composition of the TME, therefore, is essential to broaden immunotherapeutic efficacy by validating novel targets and providing rationale for combination regimens. A complementary system is the tumor-containing Foxp3-DTR mouse model in which Treg cells can be removed so immune cell inhibition can maximally be released prior to immunotherapy (236). Development of new models could benefit from physical and engineering approaches, for example, leading to: (i) unique, therapeutically exploitable physical features of TMEs (e.g., distinctive tumor cell glycocalyx, vasculature compression and stiffening of ECM) identified via PET, CT, or MRI imaging; (ii) improved drug delivery specificity and dosage via nanoparticles, bioscaffold cancer vaccines, RNAi-based systems and implantable devices; and (iii) microfluidic “organs-on-chips” (237).

*Second*, of the possible some 20 different checkpoints exemplified by LAG-3, VISTA and TIM-3, only one (PD-1/PD-L1) is currently being fully exploited, so a lot remains to be evaluated. Even the functioning of PD-1 receptors needs to be understood better since whilst PD-1 blockade would be protective against solid tumors and non-T-cell cancers, it could exacerbate certain T-cell-derived tumors (36). Non-malignant cells (e.g., adipocytes, fibroblasts, pericytes and macrophages) within the TME that mediate intercellular communication between tumor cell subpopulations and can influence cancer progression could serve as viable immune-targets (2). Indeed, “tuning” and manipulation by IFNγ-facilitated conversion of macrophages of the M2/repair-type – responsible for repairing wounds—into M1/kill-type bolstered host anti-tumor response in mouse models, slowing, or stopping tumor growth (238). A meta-analysis of 90 immune checkpoint inhibitor trials found higher irAE incidence for anti-CTLA-4 (~54%), compared with anti-PD-1 (~27%) and anti-PD-L1 (~17%) (239). This would predict that checkpoint blockades targeting exclusively immune cells will have significantly higher irAE rates relative to those additionally targeting tumor cells (anti-PD-L1) (239). Future immunotherapy trial designs, therefore, should be adjusted accordingly.

*Third*, the issue of tumor resistance to immunotherapies needs in-depth understanding. Emerging evidence depicts tumor cells as “communities” or “rogue organs” with complex local population dynamics that produce both positive (commensalism and synergism) and negative (competition and parasitism) effects. To achieve long-term benefit, it is necessary to take into account both intra-tumoral heterogeneity and dynamics including treatment-induced changes (240). Patients with complete radiographical and pathological remission can still experience relapse due to minimal residual disease (MRD), especially with acute leukemia and aggressive lymphomas (241). In fact, instead of waiting for patients to relapse after effective therapy, MRD can be detected by flow cytometry then iteratively sampled and tested to establish its therapeutic susceptibility and optimal therapies could then be administered, even indefinitely (241). However, there is the inherent risk of cost and toxicity from overtreatment or incorrect treatment (241).

*Fourth*, gut microbiota has been implicated in regulating both immunotherapy responses and carcinogenesis (242). In murine models, *Bifidobacterium* was shown to induce anti-tumor immunity and enhance the therapeutic efficacy of PD-L1 blockade (243). Different mice displayed different microflora compositions, however, even when their genetic backgrounds were identical. Thus, identifying the most therapeutically favorable microbiota for different cancer types would seem a worthwhile effort (242). In another study, prevalent commensal communities of *Bifidobacterium longum, Enterococcus faecium*, and *Collinsella aerofaciens* were found in the 38% of metastatic melanoma patients responsive to PD-1 blockade, but were less abundant in the non-responsive patients (244). Koh et al. established a strong correlation between melanoma patient response to nivolumab and gut microbiota (245). Optimally responding patients had significant intestinal tract communities of *Holdemania filiformis, Faecalibacterium prausnitzii*, and *Bacteroides thetaiotaomicron*. Additionally, lung and kidney cancer patients treated with antibiotics saw nullified PD-1 blockade efficacy. The bacterium *Akkermansia muciniphila* was found in 60–70% of PD-1 blockade-treated patients with a PR or stable disease respectively, compared with only 34% in unresponsive patients (246). Thus, probiotic cocktails may ultimately be combined with immunotherapy in a personalized setting (245).

*Fifth*, further combination therapies are possible. For example, after surgical removal of a primary malignant tumor, there is still risk of recurrence including regrowth of micro-metastases seeded from residual circulating tumor cells (CTCs) (247). Wang et al. conjugated platelets to an anti-PD-L1 checkpoint inhibitor and injected these intravenously into mouse 4T1 breast cancer model (247). Engineered platelets recruited the most immune cells to the surgery sites and thus delivered anti-PD-L1 with much greater accuracy and no significant irAE. Relative to controls, treated mice showed significantly enhanced survival. The technology is expected to find increasing applications, especially after optimizing issues like timing, dosage, and duration of combination regimens in relation to different tumor types (32, 248). In such optimization, for example, excessive elimination of immunosuppressive Treg cells, that would risk autoimmune reactions, should be avoided (249). Grinberg-Bleyer et al. recently showed that chemical ablation of c-Rel (a subunit of NF-kB transcription factor) via the muscle pain “dampener” pentoxifylline delayed melanoma development in murine models by repressing Treg-mediated immunosuppression (249). c-Rel inhibition boosted the efficacy of PD-1 blockade, allowing complete response rate in ~20% of cases. Consequently, c-Rel inhibitors may become a standard component of future immune checkpoint blockade combinations (249). Another type of combination could involve natural products. For example, an extract from the Ayurvedic herb *Andrographis paniculata* was recently identified as a potent enhancer of NK cell activity *in vitro* and *in vivo* (250). High-throughput screening techniques promise to speed up identification of possible natural immunomodulators for combination immmunotherapy (251). Obesity-related factors, including insulin-like growth factor 1, adipokines and inflammation-mediators involved in systemic metabolic function, can directly interact with tumor cell metabolics (252). Consequently, obesity contributes to negative prognosis across many cancers by influencing tumor response to immunotherapy, and promoting tumor initiation and progression. Future combinations should, therefore, be increasingly guided by the personalized physiology of patients. Furthermore, introducing repurposed non-cancer drugs into immunotherapy could pay dividend. For example, diclofenac, an affordable, and readily accessible pain killer for rheumatoid arthritis, has yielded improved efficacies with chemotherapy and radiotherapy (253). The next leap in efficacy (possibly up to ~80% patient survival rate) is anticipated to come from novel combination refinements, by embracing innovative biomarker-driven synergies and sequencing tumors using “The Cancer Genome Atlas.” This promises to significantly improve our understanding of the molecular basis of tumor immunosuppressive mechanisms and could minimize irAEs (254, 255).

*Sixth*, rapidly advancing gene-editing technologies are also applicable to immunotherapy. PD-1 gene knockout by electroporation of sgRNA:Cas9 plasmids significantly repressed PD- 1 expression without compromising human T-cell viability *in vitro*. This technique efficiently disrupted PD-1 and proved markedly more efficient in its use of electroporation, relative to current viral-based gene transfer approaches (256). The efficacy of ACT cellular therapies can also be improved by applying the same approach (256). Genetic engineering of NK cells for immunotherapy has historically been marred by low transduction efficiencies. Recently, however, mRNA electroporation successfully reprogrammed multiple NK modalities at high efficiency and reproducibility, without compromising cellular viability or phenotype (257). New efficacious avenues for adoptive NK-cell immunotherapy efficacy are thus opened, with potential to enhance many aspects of tumor targeting *in vivo* (257). Nanoparticles with gene therapeutics also offer hope. For example, mixing foxo1 mRNA nanocarriers with CAR-T cells to reprogram them, via “hit-and-run” transient expression, into more aggressive, higher efficacy and longer-lasting memory T-cells (258). Loss-of-function mutations alter the extent of malignant cells' vulnerability to T-lymphocyte-based immunotherapy (259). Patel et al. knocked-out all known protein-encoding genes across the human genome by utilizing a genome-scale CRISPR-Cas9 library and profiling all genes where functional loss induced reduced CD8+ T-cell effector function (259). Identifying these genes could then serve as a “blueprint” to establish, at a genetic level, why certain patient tumor subsets are resistant to immunotherapies (259).

*Seventh*, advanced technology and artificial intelligence also promise to contribute significantly to the next wave of developments in immune-oncology. Existing supercomputers can facilitate (i) “big data” genomic analysis to uncover patient risk factors, (ii) optimizing dosages of combination drugs, (iii) modeling tissues, cells, and drug interactions to identify novel medicines, and (iv) discovery of therapeutically exploitable relationships within complex cellular networks (260). Quantum computing, with superior processing power and speed, also promises to bring unprecedented sub-atomic detail to our understanding of immunotherapeutic effects (261).

Finally, we should stress that significant safety concerns remain in both mono- and combination immunotherapies, and much more work is required to limit and control the undesirable side effects (34, 35, 262–264). As well as pre-determining the subpopulations of patients likely to benefit from given treatment regimens, therefore, personalized medication should incorporate predictive biomarkers for identifying possible autoimmune response risk (262).

## Author contributions

HM performed the literature search supported by MD. HM wrote the main text and prepared the illustrations. MD and HM finalized the manuscript.

### Conflict of interest statement

MD is involved in a small biotechnology company aiming to exploit the anti-cancer potential of VGSCs. The remaining author declares that the research was conducted in the absence of any commercial or financial relationships that could be construed as a potential conflict of interest. The reviewer PL and handling Editor declared their shared affiliation.

## Abbreviations

ACT: adoptive cellular therapy
APC: antigen presenting cell
ARG: arginase
B-ALL: B-cell acute lymphoblastic leukemia
BBB: blood-brain-barrier
BiTE: Bi-specific T-cell engager
BTLA: B- and T-lymphocyte attenuator
CAF: cancer-associated fibroblast
CAR: chimeric antigen receptor
CCL2: C-C motif chemokine ligand 2
CD: cluster of differentiation
cdGMP: cyclic di-guanosine monophosphate
CDK: cyclin-dependent kinase
CML: chronic myeloid leukemia
COX2: cyclooxygenase 2
CRAC: calcium release activated channel
CRISPR: clustered regularly interspaced short palindromic repeats
CRS: cytokine release syndrome
CSC: cancer stem cell
CT: computed tomography
ctDNA: circulating DNA
CTL: cytotoxic T-lymphocyte
CTLA-4: cytotoxic T-lymphocyte-associated protein 4
CXCL12: chemokine (C-X-C motif) ligand 2
DC: dendritic cell
DCR: disease control rate
dMMR: DNA mismatch repair system
DNA: deoxyribonucleic acid
DNMT: DNA methyltransferase
ECM: extracellular matrix
EpCAM: epithelial cell adhesion molecule
FasL: Fas ligand
FGF: fibroblast growth factor
GAD: glutamate decarboxylase
GCK: glucokinase
GCN2: general control nondepressible-2
GITR: glucocorticoid-induced TNFR family-related protein
GM-CSF: granulocyte macrophage colony stimulating factor
GNS: gold nanostars
HCT: hematopoietic stem cell transplantation
HDAC: histone deacetylases
HVEM: herpes virus-entry mediator
ICD: immunogenic cell death
ICOS: inducible T-cell costimulatory
IDO, indoleamine 2: 3-dioxygenase
IFN: interferon
Ig: immunoglobulin
IKKβ: IkB-Kinase β
IL: interleukin
irAE: immune-related adverse effect
ITIM: immunoreceptor tyrosine-based inhibition motif
KINK-1: kinase inhibitor of NF-kB-1
LAG-3: lymphocyte activation gene 3 protein
LIGHT: lymphocyte activation gene 3 protein
mAbs: monoclonal antibodies
MAPK/ERK: mitogen-activated protein kinase
M-CSF/CSF1: macrophage colony-stimulating factor
MDM2/4: murine double minute 2 homolog
MDR: multidrug resistant
MDSC: myeloid-derived suppressor cell
MHC: major histocompatibility complex
MRD: minimal residual disease
MRI: magnetic resonance imaging
mRNA: messenger RNA
miRNA: micro RNA
MSI-H: microsatellite instability high
mTORC1: mammalian target of rapamycin complex 1
NCP: nano-scale coordination polymer
NFATc1: nuclear factor of activated T-cells
NF-kB: nuclear factor kB
NHL: non-Hodgkin lymphoma
NK: natural killer
NO: nitric oxide
NOTCH1: Notch homolog 1
NSCLC: non-small cell lung cancer
ORR: objective response rate
OS: overall survival
OV: oncolytic virus
PAP-1: 5-(4-phenooxybutoxy) psoralen
PD-1: programmed cell death protein 1
PDAC: pancreatic ductal adenocarcinoma
PD-L1: programmed cell death protein 1 ligand
PDT: photodynamic therapy
PDX: patient-derived xenografts
PET: positron emission tomography
PGE2: prostaglandin E2
PI3Kγ: phosphoinositide 3-kinase-γ
PSGL-1: P-selectin glycoprotein ligand-1
RCC: renal cell carcinoma
ROS: reactive oxygen species
scFv: single-chain variable fragment
Slc15a2, solute carrier family 15: member 2
STAT-3: signal transducer and activator of transcription 3
sTCR: synthetic T-cell receptor
STE: surface T-cell engagers
STING: stimulator of interferon genes
SYMPHONY: synergistic immuno photodermal nanotherapy
T-ALL: T-cell acute lymphoblastic leukemia
TAM: tumor-associated macrophage
TCGA: the cancer genome atlas
TCR: T-cell receptor
TEX: tumor-derived exosomes
TGF: transforming growth factor
TIGIT: T-cell immunoreceptor with Ig and ITIM domains
TIL: tumor infiltrating lymphocyte
TIM-3: T-cell Ig mucin domain-containing 3
TKI: tyrosine kinase inhibitor
TME: tumor microenvironment
TNBC: triple negative breast cancer
TNF: tumor necrosis factor
TNFR: tumor necrosis factor receptor
TNFRSF4 (OX40): tumor necrosis factor receptor superfamily member 4
Treg: regulatory T cell
T-VEC: talimogene laherparepvec
VEGF: vascular endothelial growth factor
VEGFR: vascular endothelial growth factor receptor
VGSC: voltage-gated sodium channel
VISTA: V-domain Ig suppressor of T-cell activation
ZIKV: Zika virus
ZnP: Zn-pyrophosphate.
