# Supplementary material for: Immuno-Oncology: Emerging Targets and Combination Therapies
Source: Front Oncol. 2018 Aug 23;8:315. doi: 10.3389/fonc.2018.00315 (PMC6115503; doi:10.3389/fonc.2018.00315)
Supplement: Supplementary file 1 [file Data_Sheet_1.PDF]

Immuno-oncology: Emerging targets and combination therapies

Henry T. Marshall* and Mustafa B. A. Djamgoz

Department of Life Sciences, Neuroscience Solutions to Cancer Research Group, Imperial College London, Sir Alexander Fleming Building, South Kensington Campus, London, SW7 2AZ, UK

*Corresponding author:
Henry Marshall
Tel: +44 (0) 207 594 5370
Fax: +44 (0) 207 594 2290
Email: henry_marshall@me.com

Number of figures: 13
Number of tables: 2
Word count: 11761

Running title: **Immunotherapy**

SUPPORTING INFORMATION

Supporting Figures

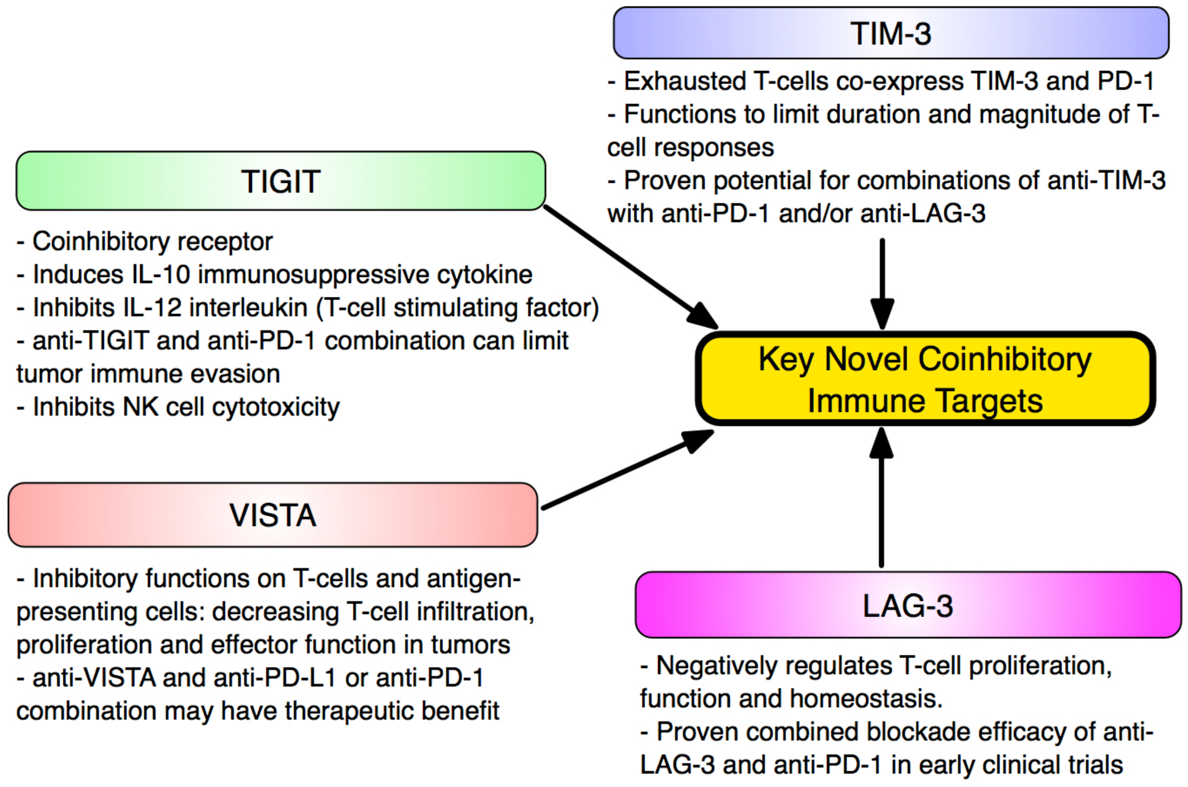

Supplementary Figure 1. Emerging and novel immunomodulatory co-targets. Key novel co-inhibitory pathways are depicted. The approach of checkpoint blockade is rapidly expanding beyond the blocking of PD-1/PD-L1 and CTLA-4 in an attempt to improve the proportion of patients responsive to immunotherapy and overcome tumor resistance. TIM-3, LAG-3 and TIGIT belong to the same receptor class as CTLA-4 and PD-1, and are among the most anticipated of the emerging checkpoint blockade targets given their proven potential for high efficacy and improved safety profile *vs* CTLA-4 and PD-1. Created using information from Baumeister et al. (1), Anderson et al. (2), Dempke et al. (3), and Marin-Acevedo et al. (4). See Abbreviations list for further definitions.

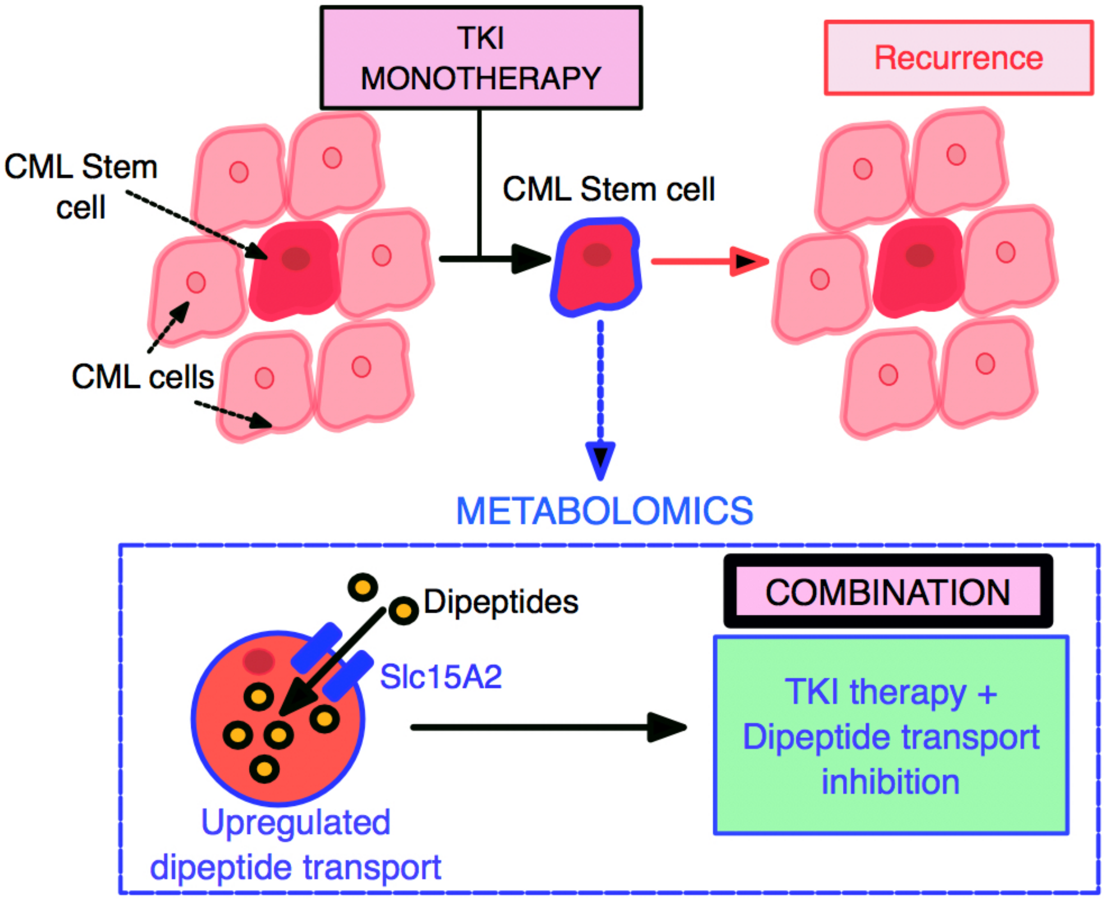

Supplementary Figure 2. Novel synergistic combinations against cancer metabolism: TKIs and dipeptide transport inhibition. Tumor metabolism has significant impact on immune responses. Metabolomics identified a key signature underpinning rare CML stem cell maintenance, and responsible for recurrence in CML patients. This directly led to the development of a novel synergistic combination of Slc15A2 dipeptide transporter inhibitor plus TKI, that will likely improve response durability in CML patients. CML, chronic myeloid leukaemia; Slc15a2, solute carrier family 15, member 2; TKI, tyrosine-kinase inhibitor. Adapted from Naka et al. (5).

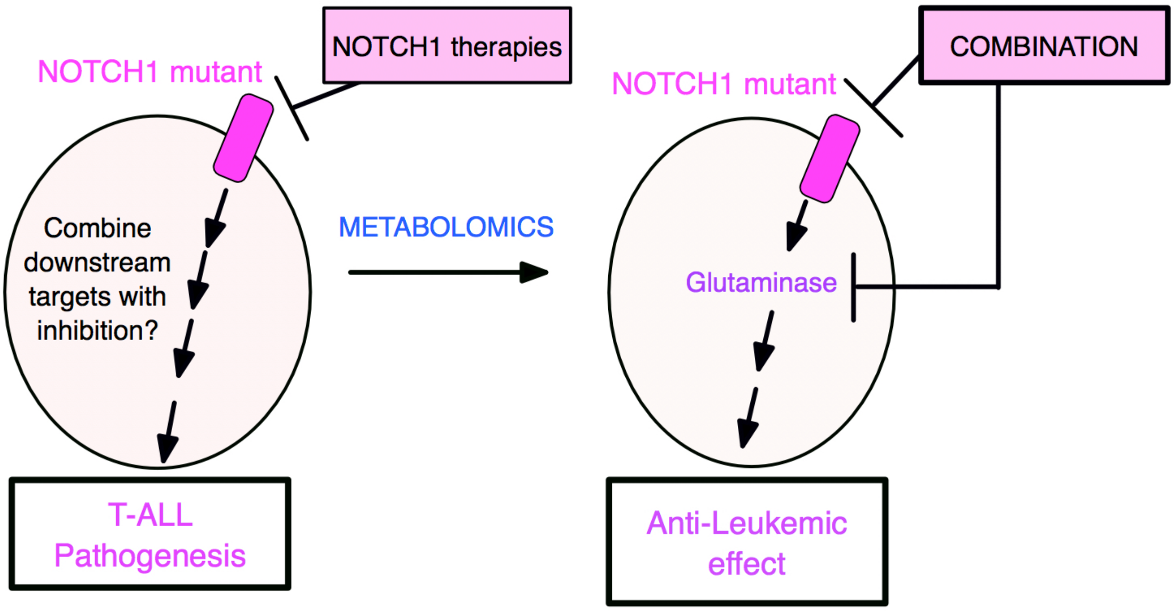

Supplementary Figure 3. Novel NOTCH1 and glutaminase inhibitor combination. In excess of 60% of T-ALL cases occur with accompanying activating mutations in NOTCH1 receptors. Metabolomics identified glutaminolysis as a pathway critical to leukemia progression downstream of NOTCH1. Anti-glutaminase plus anti-NOTCH1 yielded potent synergistic effects in patient derived T-ALL murine models. T-ALL, T-cell acute lymphoblastic leukemia; NOTCH1, notch homolog 1 translocation-associated (Drosophilia). Adapted from Herranz et al. (6).

REFERENCES

1. Baumeister SH, Freeman GJ, Dranoff G, Sharpe AH. Coinhibitory Pathways in Immunotherapy for Cancer. *Annu Rev Immunol* (2016) **34**:539–573. doi:10.1146/annurev-immunol-032414-112049
2. Anderson AC, Joller N, Kuchroo VK. Lag-3, Tim-3, and TIGIT: Co-inhibitory Receptors with Specialized Functions in Immune Regulation. *Immunity* (2016) **44**:989–1004. doi:10.1016/j.immuni.2016.05.001
3. Dempke WCM, Fenchel K, Uciechowski P, Dale SP. Second- and third-generation drugs for immuno-oncology treatment—The more the better? *Eur J Cancer* (2017) **74**:55–72. doi:10.1016/j.ejca.2017.01.001
4. Marin-Acevedo JA, Dholaria B, Soyano AE, Knutson KL, Chumsri S, Lou Y. Next generation of immune checkpoint therapy in cancer: new developments and challenges. *J Hematol Oncol* (2018) **11**:39. doi:10.1186/s13045-018-0582-8
5. Naka K, Jomen Y, Ishihara K, Kim J, Ishimoto T, Bae E-J, Mohney RP, Stirdivant SM, Oshima H, Oshima M, et al. Dipeptide species regulate p38MAPK-Smad3 signalling to maintain chronic myelogenous leukaemia stem cells. *Nat Commun* (2015) **6**:8039. doi:10.1038/ncomms9039
6. Herranz D, Ambesi-Impiombato A, Sudderth J, Sánchez-Martín M, Belver L, Tosello V, Xu L, Wendorff AA, Castillo M, Haydu JE, et al. Metabolic reprogramming induces resistance to anti-NOTCH1 therapies in T cell acute lymphoblastic leukemia. *Nat Med* (2015) **21**:1182–9. doi:10.1038/nm.3955
